# Time-Dependent Increase in Susceptibility and Severity of Secondary Bacterial Infections During SARS-CoV-2

**DOI:** 10.3389/fimmu.2022.894534

**Published:** 2022-05-12

**Authors:** Amanda P. Smith, Evan P. Williams, Taylor R. Plunkett, Muneeswaran Selvaraj, Lindey C. Lane, Lillian Zalduondo, Yi Xue, Peter Vogel, Rudragouda Channappanavar, Colleen B. Jonsson, Amber M. Smith

**Affiliations:** ^1^Department of Pediatrics, University of Tennessee Health Science Center, Memphis, TN, United States; ^2^Department of Microbiology, Immunology and Biochemistry, University of Tennessee Health Science Center, Memphis, TN, United States; ^3^Department of Acute and Tertiary Care, University of Tennessee Health Science Center, Memphis, TN, United States; ^4^College of Pharmacy, University of Tennessee Health Science Center, Memphis, TN, United States; ^5^Animal Resources Center and Veterinary Pathology Core, St. Jude Children’s Research Hospital, Memphis, TN, United States; ^6^Institute for the Study of Host-Pathogen Systems, University of Tennessee Health Science Center, Memphis, TN, United States

**Keywords:** SARS-CoV-2, COVID-19, Streptococcus pnemoniae, pneumococcus, immune response, coinfection

## Abstract

Secondary bacterial infections can exacerbate SARS-CoV-2 infection, but their prevalence and impact remain poorly understood. Here, we established that a mild to moderate infection with the SARS-CoV-2 USA-WA1/2020 strain increased the risk of pneumococcal (type 2 strain D39) coinfection in a time-dependent, but sex-independent, manner in the transgenic K18-hACE2 mouse model of COVID-19. Bacterial coinfection increased lethality when the bacteria was initiated at 5 or 7 d post-virus infection (pvi) but not at 3 d pvi. Bacterial outgrowth was accompanied by neutrophilia in the groups coinfected at 7 d pvi and reductions in B cells, T cells, IL-6, IL-15, IL-18, and LIF were present in groups coinfected at 5 d pvi. However, viral burden, lung pathology, cytokines, chemokines, and immune cell activation were largely unchanged after bacterial coinfection. Examining surviving animals more than a week after infection resolution suggested that immune cell activation remained high and was exacerbated in the lungs of coinfected animals compared with SARS-CoV-2 infection alone. These data suggest that SARS-CoV-2 increases susceptibility and pathogenicity to bacterial coinfection, and further studies are needed to understand and combat disease associated with bacterial pneumonia in COVID-19 patients.

## Introduction

Throughout the coronavirus disease 2019 (COVID-19) pandemic caused by the severe acute respiratory syndrome coronavirus 2 (SARS-CoV-2), there have been case reports, multi-center cohort studies, systematic reviews, and meta-analyses assessing the extent and severity of coinfections with secondary pathogens including viruses, fungi, and bacteria (1–31). Although coinfection rates varied across studies, some studies suggested that coinfecting respiratory bacteria were predictors of severe SARS-CoV-2-related disease and mortality (23–31). Bacterial pathogens that were detected included *Mycoplasma pneumoniae*, *Legionella pneumophila*, *Chlamydophila pneumoniae*, *Klebsiella pneumoniae*, *Pseudomonas aeruginosa*, *Haemophilus influenzae*, *Acinetobacter baumanii, Staphylococcus aureus*, and *Streptococcus pneumoniae* (pneumococcus). Pneumococcus, which is a major cause of community-acquired pneumonia (32–34), was detected by throat swab in 0.8% (8) to 7.2% (5) of hospitalized COVID-19 patients not requiring intensive care unit (ICU) admission or invasive respiratory support, while the frequency tended to be higher [6.5% (24) to 59.5% (4)] in patients with severe respiratory distress. Because bacterial transmission has largely been dampened by non-pharmaceutical measures (e.g., masking and physical distancing), it is important to understand whether SARS-CoV-2 infection predisposes individuals to bacterial infections and, if so, what clinical and immunological changes occur as a result of coinfection.

In general, viral-bacterial coinfections are not uncommon, where *S. aureus* and pneumococcus are widely documented as complicating pathogens during infection with other viruses, most notably influenza A virus (IAV) [Reviewed in (35–46)]. During influenza pandemics, 45-95% of the mortality has been attributed to bacterial coinfections (47–50). Fortunately, the impact of these complications has appeared to be lower during the SARS-CoV-2 pandemic, but these could increase as novel variants arise and as SARS-CoV-2 becomes endemic. IAV and SARS-CoV-2 both cause infections that range from asymptomatic to severe, but SARS-CoV-2 has a longer incubation period, longer and more varied duration of viral shedding and symptoms, and more pathological effects on tissues outside of the respiratory tract [Reviewed in (51–54)]. Although viral burden does not directly correlate to disease (55–61), both viruses can induce significant lung damage [Reviewed in (52–54)]. Some host responses also differ in timing and magnitude, including the delayed type I interferon (IFN-α,β), increased proinflammatory cytokines like TNF-α and IL-6, and reduced immune regulation that have been detected in COVID-19 patients (62–66). Further, neutrophils and macrophages, which are important for efficient bacterial clearance during viral-bacterial coinfection (67–72), are dysregulated during COVID-19 (73–75). Thus, the potential for bacterial invasion during SARS-CoV-2 infection may also differ from that observed in influenza infection with respect to timing and host-pathogen mechanisms.

While the investigation of viral and immune dynamics in the lower respiratory tract is difficult to assess in humans, they have been clarified in animal models. One study using SARS-CoV-1 suggested that bacteria can enhance pathogenicity of coronaviruses (76), and numerous studies of influenza-bacterial coinfection indicate that susceptibility and pathogenicity of bacterial coinfections are time-dependent with the greatest mortality observed when bacteria is initiated at 7 d pvi (77). The progressive increase in susceptibility to bacterial coinfection during influenza is largely due to the depletion and/or dysfunction of resident alveolar macrophages (AMΦ) during IAV infection, which is dynamic throughout the infection (55, 67) and maximal at 7 d pvi (55, 67–69). Following bacterial establishment, dysfunction of neutrophils (78–81), which may be in part facilitated by bacterial metabolic interactions (82) and type I IFNs (71, 82, 83), and additional depletion of AMΦ (55) contribute to bacterial growth and coinfection pathogenesis [Reviewed in (39–41, 45, 84, 85)]. Currently, the effect of SARS-CoV-2 infection on AMΦs remains somewhat unclear, although human, murine, and *in vitro* data indicate that AMΦs become productively infected with SARS-CoV-2, leading to altered cytokine production and responsiveness (86–89). In addition, SARS-CoV-2 seems particularly adept at delaying and avoiding innate immune responses, resulting in delayed or decreased T cell responses, accumulation of neutrophils and inflammatory monocytes, and enhanced lung pathology [Reviewed in (90–93)]. IAV also has mechanisms of immune evasion [Reviewed in (94, 95)] but induces a robust CD8^+^ T cell response in the lungs that efficiently clears virus. During IAV-pneumococcal coinfection, CD8^+^ T cells are depleted (96), and viral loads rebound (55, 68, 82). Mechanisms for both of these are being investigated, but direct viral-bacterial interactions (97) that allow the virus to enter new areas of the lung in addition to a bacterial-mediated increase in virus production (55, 68, 98) contribute to the increased viral loads. However, these effects are overshadowed by the robust bacterial growth and bacterial-mediated effects on host responses. Given these potential mechanisms and the reported myeloid dysfunction (73–75), delayed IFN responses (62–66), and CD8^+^ T cell depletion (99–103) during SARS-CoV-2, a better understanding of the potential for bacterial invasion and the effects of coinfection on immune cell, viral, and pathological dynamics is needed and the focus of this study. To assess bacterial susceptibility during COVID-19 and determine whether a synergism exists between SARS-CoV-2 and pneumococcus, we infected K18-hACE2 mice with a low dose of SARS-CoV-2 to initiate a mild-moderate infection and coinfected the animals 3, 5, or 7 days later with pneumococcus. Bacteria were unable to establish at 3 d post-virus infection (pvi), but coinfections at 5 or 7 d pvi resulted in increased lethality in a sex-independent manner. Although viral dynamics and lung pathology were unchanged within the first 24 h of coinfection, select immune cells and proinflammatory cytokines were decreased in the lungs of animals coinfected at 5 d pvi but not at 7 d pvi. These findings support the increased susceptibility of SARS-CoV-2-infected individuals to bacteria and highlight numerous distinct features from other viral-bacterial coinfections.

## Results

### Time-Dependent Increases in Lethality During SARS-CoV-2-Pneumococcal Coinfection

**Figure 1:**
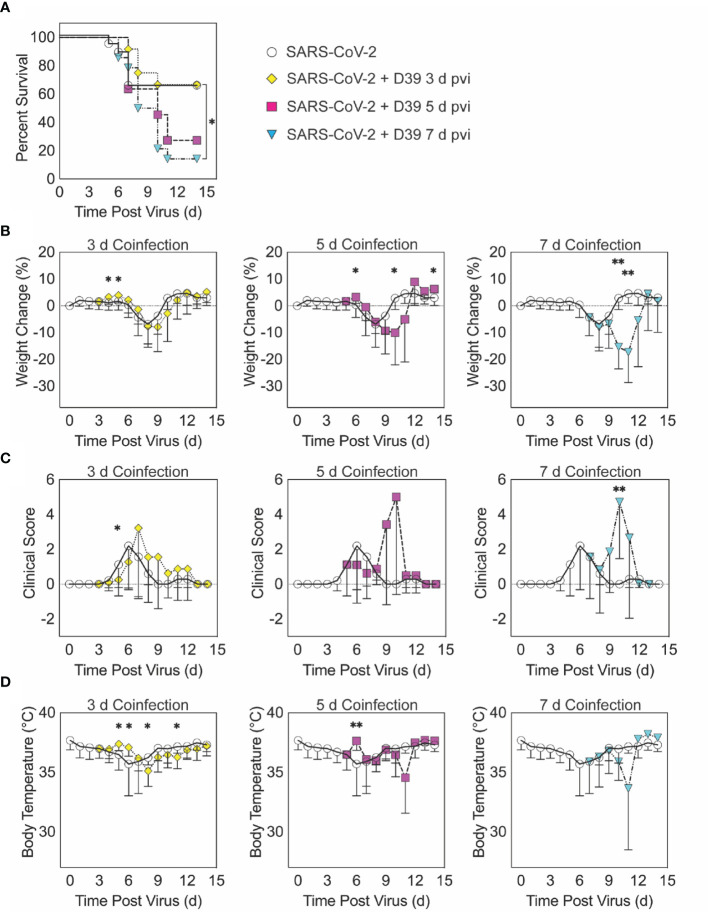
SARS-CoV-2-pneumococcal coinfection in K18-hACE2 mice. Kaplan-Meier survival curves **(A)**, percent weight loss **(B)**, cumulative clinical score **(C)**, and temperature **(D)** of mice infected with SARS-CoV-2 (250 PFU; white circles, solid lines) followed by 10^3^ CFU D39 at 3 d (yellow diamonds, dotted lines), 5 d (magenta squares, dashed lines), or 7 d (cyan triangles, dash-dotted lines) pvi. Data are shown as the mean ± standard deviation (SD) and significant differences are indicated by *,*P* < 0.05; **,*P* < 0.01 for comparisons between SARS-CoV-2 infection and SARS-CoV-2-pneumococcal coinfection.

To examine the susceptibility and pathogenicity of pneumococcus coinfection during SARS-CoV-2 infection, K18-hACE2 mice (male and female, 10 to 13 weeks old) were infected with 250 PFU of SARS-CoV-2 or PBS followed by 10^3^ CFU of pneumococcal strain D39 (coinfected) or PBS (mock coinfected) at either 3, 5, or 7 d pvi. During mock coinfection, the selected viral dose was lethal in 35% of mice (**Figure 1A**) and caused weight loss from 5 to 11 d pvi with maximum weight loss (average 7%) at 8 d pvi (**Figure 1B**) and clinical scores peaking at 6 d pvi (**Figure 1C**). In the absence of viral infection, the selected bacterial dose was lethal in 1/6 mice (17% lethality) at 4 d post bacterial infection (pbi) (**Figure S1A**) and caused only mild, transient weight loss (~3%) (**Figure S1B**) and increased temperatures (**Figure S1C**) after 1 to 2 d pbi.

When the bacterial coinfection was initiated at 3 d pvi, lethality was not enhanced (*P* = 0.73) (**Figure 1A**). Interestingly, weight loss in coinfected animals was reduced at 1 d (*P* = 0.03) and 2 d (*P* = 0.04) pbi (**Figure 1B**) and the cumulative clinical score was lower at 2 d pbi (*P* = 0.03) (**Figure 1C**) compared with mock coinfected controls. In addition, the temperature of coinfected animals was higher at 2 d (*P* = 0.003) and 3 d (*P* = 0.01) pbi and lower at 5 d (*P* = 0.02) and 8 d (*P* = 0.045) pbi (**Figure 1D**). A coinfection initiated at 5 d pvi was slightly more lethal than the SARS-CoV-2 infection alone, where additional mortality was observed at 5 to 6 d pbi, but this was not statistically significant (*P* = 0.14) (**Figure 1A**). The average weight loss was reduced (*P* = 0.01) and temperature was increased (*P* = 0.001) at 1 d pbi in the coinfected animals (**Figures 1B, D**). Coinfected animals lost more weight than animals infected with SARS-CoV-2 alone at 5 d pbi (*P* = 0.03) (**Figure 1B**), but no significant difference in their clinical scores was detected (**Figure 1C**). Comparatively, a coinfection at 7 d pvi was significantly more severe than SARS-CoV-2 infection alone (*P* = 0.03) and resulted in additional lethality at earlier times than the coinfection at 5 d pvi, with additional animals succumbing to the infection within 1, 3, or 4 d pbi (**Figure 1A**). Significantly more weight loss at 3 d (*P* < 0.001) and 4 d (*P* = 0.002) pbi (**Figure 1B**) and higher clinical scores at 3 d pbi (*P* = 0.01) (**Figure 1C**) occurred without altering temperature (**Figure 1D**).

### SARS-CoV-2 Coinfection Increased Bacterial Loads but Not Viral Loads

**Figure 2:**
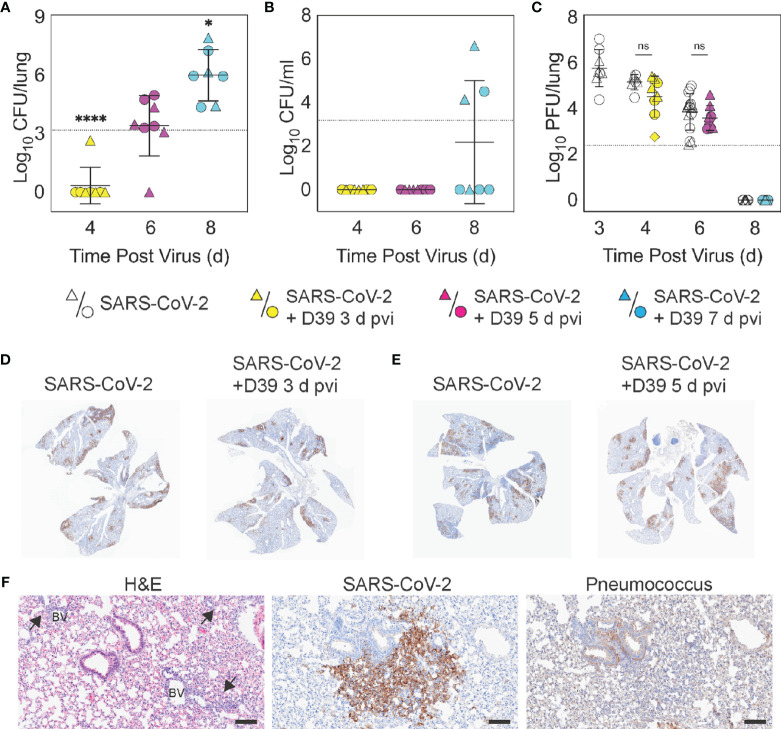
Dynamics of pathogen loads during SARS-CoV-2 infection and pneumococcal coinfection. Lung bacterial loads (CFU/lung) **(A)**, blood bacterial loads **(B)**, and lung viral loads (PFU/lung) **(C)** in female (circles) and male (triangles) mice infected with SARS-CoV-2 (250 PFU; white) followed 10^3^ CFU D39 at 3 d (yellow), 5 d (magenta), or 7 d (cyan) pvi. Each symbol represents a single mouse and the mean ± standard deviation (SD) are for combined male and female groups. Significant differences are indicated by ns, not significant; *P < 0.05; ****P < 0.0001. For bacterial titers, comparison was with the inoculum (dotted line). **(D, E)** Representative immunohistochemical (IHC) staining for SARS-CoV-2 nucleocapsid protein in whole lung sections following (24 h pbi) infection with SARS-CoV-2 (250 PFU) then PBS or 10^3^ CFU D39 at 3 d **(D)** or 5 d **(E)** pvi. **(F)** Representative lung sections stained with H&E, SARS-CoV-2 nucleocapsid protein, or pneumococcus from infection with SARS-CoV-2 (250 PFU) followed by 10^3^ CFU D39 at 5 d pvi. Lesions with perivascular inflammatory cell infiltration are indicated by arrows; blood vessel (BV). Scale bar = 100 µm.

To evaluate whether SARS-CoV-2-bacterial coinfection alters pathogen burden, we measured viral loads in the lung and bacterial loads in the lung and blood of infected animals. In mice infected with bacteria alone or with SARS-CoV-2 followed by bacteria at 3 d pvi, no bacteria were recovered from the lungs of 7/8 mice at 24 h pbi (**Figures 2A**, **S1D**). However, when the bacteria was introduced at 5 d pvi, bacterial loads in the lung remained at a level similar to the inoculum in 7/8 mice and was cleared in 1/8 mice (**Figure 2A**). Bacteria were not detected in the blood of mice infected with bacteria alone (data not shown) or SARS-CoV-2-bacteria coinfected at 3 or 5 d pvi (**Figure 2B**). However, in mice coinfected at 7 d pvi, significant bacterial growth occurred in the lungs of all animals (*P* = 0.02; Mann-Whitney test) and the blood of some animals (3/7) with titers reaching 4.4 to 7.9 log_10_ CFU/lung (**Figure 2A**) and 4.1 to 6.6 log_10_ CFU/mL (**Figure 2B**), respectively, within 24 h pbi.

Pulmonary viral loads were unchanged by bacterial coinfection whether coinfection was initiated at 3 d (*P* = 0.12) or 5 d (*P* = 0.18) pvi (**Figure 2C**) and the amount and distribution of viral antigen in the lung tissue were also unchanged (**Figures 2D, E**). Although some areas of the lung contained colocalized virus and bacteria, both intracellular and extracellular bacterial antigen were detected in areas containing no viral antigen (**Figure 2F**). The virus had cleared by 8 d pvi in the groups that were mock coinfected or bacterial coinfected at 7 d pvi (**Figure 2C**). No significant differences were found in viral or bacterial loads between males and females.

### Select Changes in Pulmonary Immune Responses After SARS-CoV-2-Pneumococcal Coinfection

**Figure 3:**
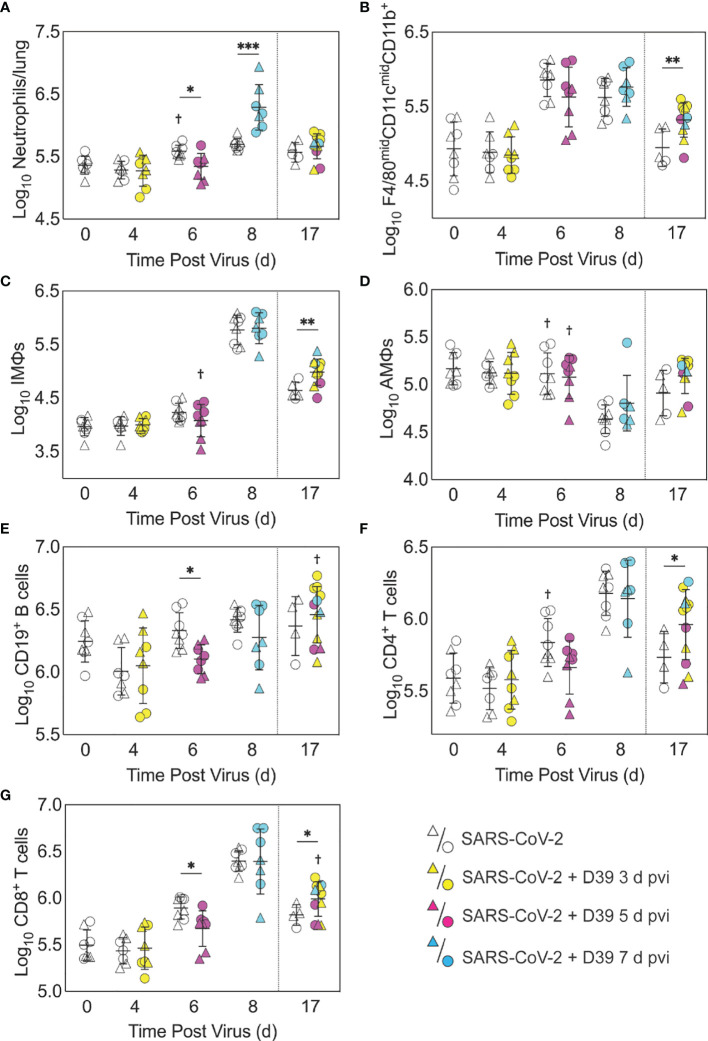
Immune cell dynamics during SARS-CoV-2 infection and pneumococcal coinfection. Total neutrophils **(A)**, F4/80^mid^CD11c^mid^CD11b^+^ monocytes/macrophages **(B)**, inflammatory macrophages (iMΦ) (F4/80^hi^CD11c^hi^CD11b^+^) **(C)**, alveolar macrophages (AMΦ) (F4/80^hi^CD11c^hi^CD11b^-^MHC-II^low/-^) **(D)**, CD19^+^ B cells **(E)**, CD4^+^ T cells **(F)**, and CD8^+^ T cells **(G)** in the lungs of female (circles) and male (triangles) mice infected with SARS-CoV-2 (250 PFU; open symbols) followed by 10^3^ CFU D39 at 3 d (yellow), 5 d (magenta), or 7 d (cyan) pvi. Each symbol represents a single mouse and the mean ± standard deviation (SD) are for combined male and female groups. Significant differences are indicated by *,*P* < 0.05; **,*P* < 0.01; ***,*P* < 0.001 for comparisons between indicated groups and by ^†^,*P* < 0.05 for differences between males and females within a group or between coinfection times within 17 d group.

**Figure 4:**
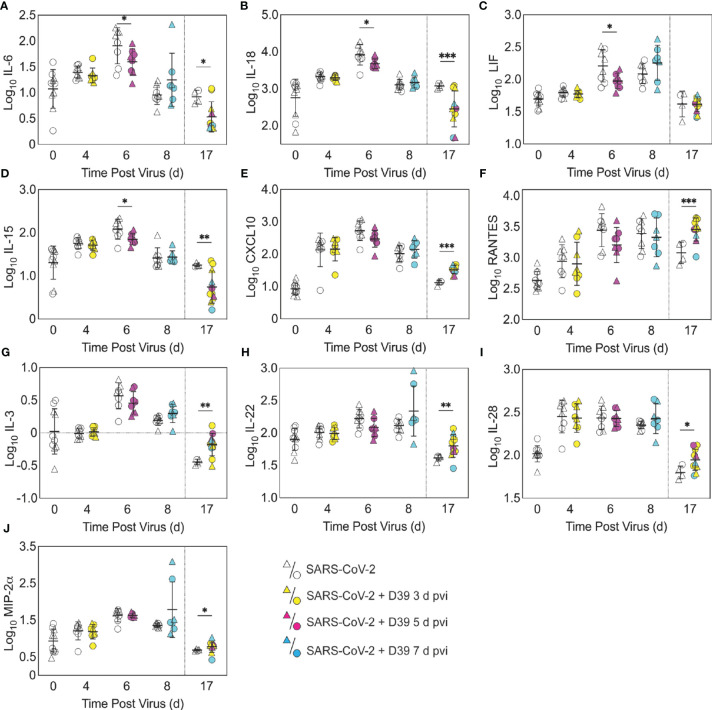
Pulmonary cytokines and chemokines during SARS-CoV-2 infection and SARS-CoV-2-pneumococcal coinfection. Total IL-6 **(A)**, IL-18 **(B)**, LIF **(C)**, IL-15 **(D)**, CXCL10 **(E)**, RANTES **(F)**, IL-3 **(G)**, IL-22 **(H)**, IL-28 **(I)**, and MIP-2α **(J)** in the lungs of female (circles) and male (triangle) mice infected with SARS-CoV-2 (250 PFU; white) followed by infection with 10^3^ CFU D39 at 3 d (yellow), 5 d (magenta), or 7 d (cyan) pvi. Each symbol represents a single mouse and the mean ± standard deviation (SD) are for combined male and female groups. Significant differences are indicated by *,*P* < 0.05; **,*P* < 0.01; ***,*P* < 0.001 for comparisons between indicated groups. Plots depicting additional cytokine and chemokine quantities (absolute log_10_ picograms) are in **Figure S5** and a heatmap representing the normalized quantity (average log_2_ change over naïve) is in **Figures S6**.

To investigate whether bacterial coinfection altered immune response dynamics, several immune cells, cytokines, and chemokines were quantified in the lung 24 h after mock coinfection or bacterial coinfection in SARS-CoV-2 infected mice (**Figures 3**, **4**, **S3, S6**). In animals infected with SARS-CoV-2 only, natural killer (NK) T cells (**Figure S3D**) and total CD19^+^ B cells (**Figure 3E**) were reduced at 4 d pvi compared with naïve (*P* = 0.007 and *P* = 0.018, respectively). The absolute numbers of other cells were unchanged at this time point (**Figures 3**, **S3**); however, increases in the proportion of activated (CD69^+^) immune cells were evident (**Figure S4**). SARS-CoV-2 infection also resulted in many cytokines and chemokines above baseline levels (all *P* < 0.05) throughout the infection, including IFN-γ, IL-1β, IL-4, IL-28, CXCL10, GM-CSF, LIF, CCL2, CCL7, MIP-1α, MIP-1β, RANTES, IFN-α, and IFN-β. IL-5, IL-6, IL-15, IL-18, M-CSF, and TNF-α were elevated at both 4 d and 6 d pvi while CXCL5, CXCL1, G-CSF, IL-3, IL-13, and IL-17A were increased only at 6 d pvi. MIP-2α, IL-2, and IL-22 were elevated at 6 d and 10 d pvi, and increased IL-10 and IL-23 were detected only at 8 d pvi (absolute values of cytokines are in **Figure 4**, **S5**; log_2_ changes over naïve in **Figure S6**).

As expected, a significant influx of CD45^+^ immune cells was evident at 6 and 8 d pvi in animals infected with SARS-CoV-2 only (both *P* < 0.001) (**Figure S3A**), including neutrophils (Ly6G^hi^; both *P* < 0.01; **Figure 3A**), the F4/80^mid^CD11c^mid^CD11b^+^ monocyte/macrophage subset (both *P* < 0.001; **Figure 3B**), inflammatory macrophages (F4/80^hi^CD11c^hi^CD11b^+^, iMΦ; *P* = 0.02 and *P* < 0.001, respectively; **Figure 3C**), F4/80^mid^CD11c^-^ cells (both *P* < 0.001; **Figure S3B**), NK cells (both *P* < 0.001; **Figure S3C**), CD4^+^ T cells (*P* = 0.02 and *P* < 0.001, respectively; **Figure 3F**), and CD8^+^ T cells (both *P* < 0.001; **Figure 3G**). Unlike the pathogen loads, some of the immune cells were different between males and female that were mock coinfected at 5 d pvi, including neutrophils (*P* = 0.047), resident alveolar macrophages (F4/80^hi^CD11c^hi^CD11b^-^MHC-II^low/-^, AMΦ; *P* = 0.047), CD4^+^ T cells (*P* = 0.02), NK cells (*P* = 0.03), and NK T cells (*P* = 0.02), which were higher in females than males.

In the groups coinfected with bacteria at 3 d pvi, no changes were observed in the absolute number (**Figures 3**, **S3**) or activation (**Figure S4**) of any quantified immune cell subset or the amount of cytokines and cytokines (**Figures 4**, **S5**) within 24 h pbi compared with mock **c**oinfection. A bacterial coinfection at 5 d pvi resulted in fewer total CD45+ cells (*P* = 0.03; **Figure S3A**), including neutrophils (**Figure 3A**), CD19^+^ B cells (**Figure 3E**), CD8^+^ T cells (**Figure 3G**), and F4/80^mid^CD11c^-^ cells (**Figure S3B**) (all *P* < 0.05) compared with the mock coinfected groups. In addition, iMΦ (*P* = 0.01) and AMΦ (*P* = 0.047) were again higher in females than males following coinfection at 5 d pvi (**Figures 3C, D**). The extent of activation was not different between the mock coinfection and bacterial coinfection at 5 d pvi (**Figure S4**), but reduced IL-6, IL-18, LIF (all *P* = 0.04), and IL-15 (*P* = 0.02) was observed at 24 h pbi (**Figures 4A-D**).

Coinfection at 7 d pvi induced a significant increase in neutrophils at 24 h pbi (*P* < 0.001) (**Figure 3A**) without altering the number or activation of any other immune cell quantified (**Figures 3**, **S3, S4**). AMΦ were reduced in the mock coinfected group compared with naïve animals (*P* = 0.001) but were not different between the mock coinfection and bacterial coinfection (*P* = 0.29) (**Figure 3D**). Absolute cell numbers and activation did not differ between male and female mice following coinfection at 7 d pvi (**Figures 3**, **S3, S4**). Perhaps unexpectedly, none of the measured cytokines were significantly different between animals that were mock coinfected and animals that were bacterial coinfected at 7 d pvi (**Figure 4** and **Figure S5**).

### Pneumococcal Coinfection Resulted in Sustained Increases in Pulmonary Immune Responses After Recovery

To investigate whether bacterial coinfection altered immune cell dynamics and activation in recovered animals, pulmonary immune cells, cytokines, and chemokines were quantified at 17 d pvi following mock coinfection or bacterial coinfection at 3, 5, or 7 d pvi. The number of iMΦ (*P* = 0.01) (**Figure 3C**) and CD8^+^ T cells (*P* = 0.02) (**Figure 3G**), as well as the activated proportion of iMΦ (*P* = 0.004), CD8^+^ T cells (*P* = 0.001), CD4^+^ T cells *P* 0.001), and CD19+ B cells (*P* = 0.005) (**Figure S4**), remained increased above naïve levels in the lungs of animals that recovered from SARS-CoV-2 infection alone. These changes were accompanied by elevated IFN-γ, CXCL10, and RANTES (*P* = 0.01, *P* = 0.03, and *P* = 0.04, respectively) at 17 d pvi compared to naïve (**Figures 4**, **S5, S6**). However, many measured cytokines and chemokines were below naive levels at 17 d pvi in the lungs of animals infected with SARS-CoV-2 only, including eotaxin, IL-2, IL-3, IL-17A, IL-22, IL-27, IL-28, M-CSF, and MIP-2α (all *P* < 0.05) (**Figures 4**, **S5, S6**).

A sustained increase in immune cell accumulation and activation was evident in animals that recovered from SARS-CoV-2-pneumococcal coinfection. At 17 d pvi, an increased absolute number and activated proportion of F4/80^mid^CD11c^mid^CD11b^+^ monocytes/macrophages (*P* = 0.01; **Figures 3B**, **4B**), iMΦ (*P* = 0.01; **Figures 3C**, **S4C**), and CD4^+^ and CD8^+^ T cells (*P* = 0.03 and 0.02, respectively; **Figures 3F, G**, **S4F, G**) were present in coinfected mice compared with mock coinfected mice. Comparison between the coinfected groups indicated that more CD8^+^ T cells were present at 17 d pvi in mice that were coinfected at 3 d or 7 d pvi than those coinfected at 5 d pvi (both *P* = 0.02; **Figure 3G**). In addition, animals that recovered from a coinfection at 7 d pvi had more activated neutrophils or iMΦ than those who recovered from a coinfection at 3 d pvi (*P* = 0.04) or 5 d pvi (*P* = 0.03), respectively (**Figures S4A, C**). These changes were accompanied by higher levels of CXCL-10 (*P* < 0.001), MIP-2α (*P* = 0.04), IL-3 (*P* = 0.001), IL-22 (*P* < 0.008), IL-28 (*P* = 0.01), and RANTES (*P* < 0.001) in the lungs of mice that had recovered from a bacterial coinfection compared with those recovered from SARS-CoV-2 alone (17 d pvi; **Figure 4E–J**). In addition, select cytokines and chemokines were reduced in animals that recovered from bacterial coinfection compared with those that were mock coinfected, including CXCL-1 (*P* = 0.01), IL-1α (*P* = 0.04), IL-6 (*P* = 0.03), IL-9 (*P* = 0.03), IL-10 (*P* < 0.001), IL-13 (*P* < 0.001), IL-15 (*P* = 0.001), IL-18 (*P* < 0.001), G-CSF (*P* = 0.03), and TNF-α (*P* =0.02) (17 d pvi; **Figures 4**, **S5**). These cytokines, except for IL-1α (*P* = 0.19) and IL-18 (*P* = 0.09), were also below baseline levels (all *P* < 0.05). In addition, IL-2 (*P* = 0.02), IL-5 (*P* = 0.02), IL-17A (*P* = 0.04), and eotaxin (*P* = 0.01) were below baseline in both the bacterial coinfected and mock coinfected groups (**Figures 4**, **S5**).

### Bacterial Coinfection Did Not Enhance Lung Pathology

**Figure 5:**
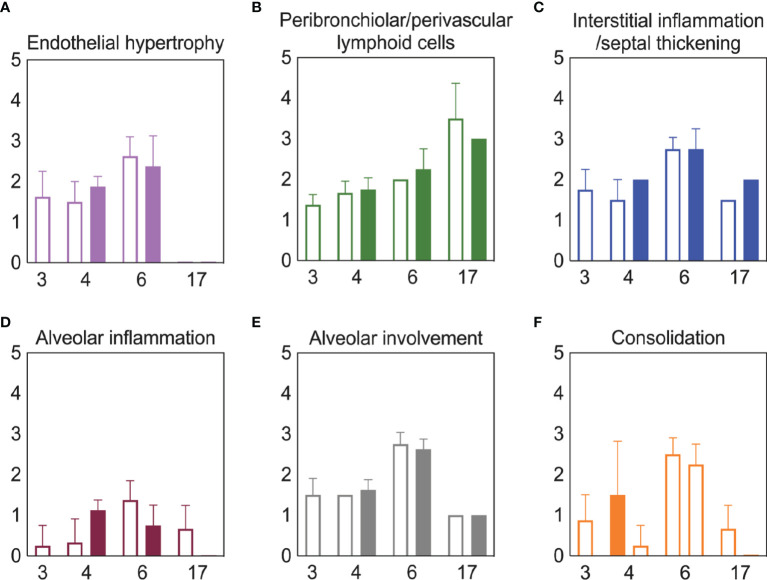
Lung pathology during SARS-CoV-2 infection and pneumococcal coinfection. Average endothelial hypertrophy **(A)**, peribronchiolar/perivascular lymphoid cells **(B)**, interstitial inflammation/septal thickening **(C)**, alveolar inflammation **(D)**, extent of alveolar involvement **(E)**, and consolidation **(F)** in lungs of mice infected with SARS-CoV-2 (250 PFU; open bars) followed by 10^3^ CFU D39 at 3 or 5 d pvi (filled bars). Plots represent the mean ± standard deviation (SD) bars for combined male and female groups.

To examine whether lung pathology was enhanced during SARS-CoV-2-pneumococcal coinfection, we assessed seven pathological features (endothelial hypertrophy/margination, peribronchiolar/perivascular lymphoid cells, interstitial inflammation/septal thickening, alveolar inflammation, alveolar edema/hemorrhage, the extent of alveolar involvement, and consolidation (**Figure 5**). There were no significant differences in any of these measurements between mock coinfected animals and those coinfected with bacteria at 3 or 5 d pvi at either 24 h pbi or 17 d pvi.

## Discussion

Currently, clinical data suggests variable, but moderate, frequency of bacterial coinfections in hospitalized COVID-19 patients (1–29). The wide range of reported rates is, at least in part, due to heterogeneous study designs, variability in the disease severity, age, and/or comorbidities of each cohort, the collection and detection methods used, and the panel of pathogens screened. Further, the reduced transmission of many pathogens (104–108) might have kept the rates of SARS-CoV-2-related bacterial pneumonia at an artificially low level during the COVID-19 pandemic. The results from this study suggest that we might expect more complications from bacterial pathogens going forward even in mild SARS-CoV-2 scenarios, which are becoming more common due to vaccine availability (109–111).

Here, we used the K18-hACE2 mouse model to establish that SARS-CoV-2 infection increases the risk of bacterial coinfection in a time-dependent manner with increased disease severity, pulmonary bacterial burden, bacteremia, and neutrophilia. This time dependency is similar to that of influenza-bacterial coinfections, but the lethality during the SARS-CoV-2-pneumococcal coinfection (**Figure 1**) was delayed comparatively (77) and some animals survived. In contrast, influenza-pneumococcal coinfections at similar doses consistently result in 100% lethality within 1-3 d pbi (77). Although further studies are needed to assess the potential for more severe coinfections at later time points, this may indicate a larger window for administration of antibacterial therapies in coinfected patients.

Mechanisms that contribute to increased risk and severity of bacterial coinfection during acute pulmonary diseases are complex and varied [Reviewed in (36, 39–41, 45, 84, 85, 112)]. While the mechanisms for SARS-CoV-2-bacterial coinfections remain unknown, the similar time-dependent susceptibility during influenza may yield insight. We and others have shown that viral-induced changes to the number (67, 69, 70) or functionality (70, 72, 113–115) of AMΦs, which may be mediated by IFN-γ (55, 115, 116), render these cells less capable of clearing bacteria. Here, SARS-CoV-2-pneumococcal coinfection did coincide with a virally induced reduction in AMΦ (**Figure 3**), which may suggest a contribution of these cells to the acquisition of bacteria during COVID-19 particularly when paired with evidence of a dysfunctional myeloid response in patients with severe infections (75). Further studies to determine how a productive SARS-CoV-2 infection of AMΦ alters infection dynamics, their production of IFN, and their phagocytic capacity (86–89) are needed. In addition, IFN-independent mechanisms of macrophage dysfunction should also be investigated because some studies suggest that RSV coinfection severity is mediated by Gas6/Axl polarization of AMΦ to non-antibacterial (M2) type cells (117). Other mechanisms, including viral-mediated changes in bacterial receptor expression and binding (77, 118–121) and the degradation of epithelial tight junction integrity (122, 123) may also promote bacterial adherence during IAV or RSV infections, and some evidence suggests that these also occur during SARS-CoV-2 infection (124–126). However, the limited colocalization of pneumococcus with SARS-CoV-2 suggests a limited role (**Figure 2**).

Several studies have found that neutrophil dysfunction contributes to pathogenicity of IAV-pneumococcal coinfection, and this seems to be mediated by bacterial metabolism (82) and type I IFNs (71, 83, 127). However, unlike IAV-pneumococcal coinfections, type I IFNs were unchanged after SARS-CoV-2-pneumococcal coinfection (**Figure S5**) and neutrophil infiltration was only observed in coinfection at 7 d pvi (**Figure 3A**), suggesting that there may be different mechanisms underlying the enhanced pathogenicity of SARS-CoV-2 pneumococcal coinfection. This may, in part, be related to the low dose used here, where some studies have found that the SARS-CoV-related alterations to the IFN and iMФ responses occur during more severe infections (128). It was intriguing to see here that cytokine production was largely unchanged at 24 h pbi (**Figures 4**, **S5**), which is in contrast with the robust proinflammatory cytokine/chemokine production during other viral-bacterial coinfections (39–41, 45, 84, 85). Perhaps unexpectedly, several cytokines associated with severe COVID-19 and damaging cytokine overproduction (IL-6, IL-15, and IL-18) (129, 130) were reduced following coinfection at 5 d pvi (**Figure 4**).

Although coinfections are typically thought to be hyperinflammatory with enhanced disease severity, tissue inflammation does not seem to be altered during SARS-CoV-2-pneumococcal (**Figure 5**) or influenza-pneumococcal (55) coinfections even with large neutrophil infiltrations (55, 82) (**Figure 3A**), at least within the first few days of coinfection. This may be owed to the nonlinearities between host immune responses, tissue inflammation, and disease severity (55, 56). Although the pathogenicity was increased during the coinfections at 5 d and 7 d pvi, there seemed to be little contribution from SARS-CoV-2, where the burden and distribution did not change within the first 24 h pbi (**Figure 2**) despite reduced CD8^+^ T cells in some groups (**Figure 3G**). In IAV-pneumococcal coinfections, invading bacteria result in robustly increased viral loads (55, 68, 82, 131–133) regardless of timing (55) and viral dissemination in the lung is increased by 30-50% (55). Our prior work (55) suggests this is due to a combination of direct viral-bacterial interactions (97) that lead to viral access to new areas of the lung in addition to increased virus production rates (68) that may be mediated by alterations to the antiviral IFN response (98). The lack of detection of SARS-CoV-2 in new areas of the lung and the lack of significant colocalization of virus and bacteria (**Figure 2**) may suggest that SARS-CoV-2 cannot as readily attach to pneumococcus like other viruses (97, 134), which is positive news given that pneumococci easily invade the blood [Reviewed in (135)] and SARS-CoV-2 affects numerous other organs (51–54).

Although the long-term effects of viral-bacterial coinfections are not well studied, these data suggest they may be important where the SARS-CoV-2-bacterial coinfection resulted in lasting immunologic changes in recovered individuals. The higher macrophages and T cells (**Figure 3**) and their associated cytokines (**Figures 4**, **S5**) at 17 d pvi in animals recovered from bacterial coinfection is intriguing and suggests sustained immunopathology (55, 56, 136, 137). Many of the elevated responses are indicators of acute respiratory distress syndrome (ARDS) (138, 139) and are upregulated to promote tissue recovery and reduce pathology (140–143). This was reflected in the slightly greater interstitial inflammation 17 d pvi (**Figure 5**) in coinfected animals. However, several cytokines were lower in animals that had recovered from bacterial coinfection with some below that of a naïve animal (**Figures S5 and S6**), which may support a remodeling environment induced, in part, by hyporesponsive epithelial cells downregulating inflammatory cytokine production to minimize local immune activation [Reviewed in (144)]. In addition, the reductions in Th2 cytokines (e.g., IL-13, IL-5, and IL-9) may be an attempt to improve lung function (145–148) while limiting hyperreactivity and further damage. Nevertheless, our results suggest a lengthy recovery of the lung from both SARS-CoV-2 and SARS-CoV-2-related secondary bacterial infections.

Vaccinating against SARS-CoV-2 is likely to prove important for reducing the incidence and severity of bacterial coinfections as it has for influenza (149). The robust efficacy of the SARS-CoV-2 vaccines is encouraging (150–153), but infection is still possible with viral replication in the nasopharynx in some vaccinated individuals (154–157). This could present an opportunity for bacterial pathogens to invade and worsen the infection. With few vaccines available for coinfecting bacteria (149), the interactions within the nasopharynx between this virus and both commensal and pathogenic bacteria will be important to study.

In summary, we used the transgenic K18-hACE2 mouse model (158) to establish that a low dose SARS-CoV-2 infection increases the risk of pneumococcal coinfection in a time-dependent manner. The data importantly highlight many differences with other viral-bacterial coinfections and the need for further studies to clarify the host-pathogen interplay that enhance susceptibility and pathogenicity during SARS-CoV-2-bacterial coinfection. This information may be crucial going forward, particularly because a sustained immune activation following coinfection suggests an increased risk of developing ARDS even in patients with mild COVID-19. In addition, as new SARS-CoV-2 variants emerge and nonpharmaceutical measures, such as wearing masks and physical distancing, become less common, we might anticipate an increase in risk of bacterial transmission and acquisition in COVID-19-infected individuals.

## Materials and Methods

### Mice

Adult (10-13 week old) male and female K18-hACE2 transgenic mice (B6.Cg-Tg(K18-ACE2)2Prlmn/J) were obtained from Jackson Laboratories (Bar Harbor, ME). Mice were housed in groups of 4 in solid–bottom polysulfone individually ventilated cages (Allentown BCU) in rooms maintained on a 12:12-hour light:dark cycle at 22 ± 2°C with 30-70% humidity in the Regional Biocontainment Laboratory (animal biosafety level 3 facility) at UTHSC (Memphis, TN). Mice were acclimated for 1 day before being lightly anesthetized with 2% inhaled isoflurane (Baxter, Deerfield, IL) and implanted subcutaneously with an IPTT300 transponder (Bio Medic Data Systems, Seaford, DE) for identification and temperature monitoring, followed by an additional 3 days of acclimation before inclusion in the experiments. Envigo irradiated rodent diet (catalog no. 7912) and autoclaved water were available ad libitum during the acclimation and study periods; gel food and hydrogel were provided at the time of infection. All experimental procedures were performed under protocol 20-0132 approved by the Institutional Animal Care and Use Committee at University of Tennessee Health Science Center (UTHSC) under relevant institutional and American Veterinary Medical Association (AVMA) guidelines and were performed in a animal biosafety level 3 facility that is accredited by the American Association for Laboratory Animal Science (AALAS).

### Infection Experiments

All experiments were done using 2019-nCoV/USA-WA1/2020 (BEI Resources NR-52281) (SARS-CoV-2) and type 2 pneumococcal strain D39. The viral infectious dose [plaque forming units (PFU)] was determined by plaque assay of serial dilutions on Vero E6 cells. Virus seed stocks were sequenced using next-generation sequencing with ARTIC primers on the Illumina MiSeq. Bacterial infectious dose [colony forming units (CFU)] was determined by using serial dilutions on tryptic soy agar plates supplemented with 3% sheep erythrocytes (TSA). Doses of virus and bacteria were selected that elicited mild-moderate disease independently to ensure that changes in disease severity following coinfection would be evident. Frozen stocks were diluted in sterile PBS and administered intranasally to groups of 4 mice, lightly anesthetized with 2.5% inhaled isoflurane (Baxter, Deerfield, IL) in a total volume of 50 µl (25 µl per nostril). Mice were inoculated with either PBS or SARS-CoV-2 at day 0 then with 10^3^ CFU of D39 or PBS, either 3 or 5 days later. Assessment of symptom severity was performed twice daily after the onset of symptoms by assigning a score (scale 0-3) to clinical features, including weight loss (0, <15%; 1, 15-20%; 2, 21-25%; 3, >25%), temperature change (0, >34°C; 1, 34-31°C; 2, 30-26°C; 3, <26°C), body condition/appearance (0, normal; 1, roughened fur; 2, roughened fur, hunched posture, mild grimace, active; 3, roughened fur, hunched posture, grimace, inactive, conjunctivitis, head-tilt), respiratory effort (0, normal; 2, increased respiratory rate and effort; 3, weak, intermittent breathing), behavior (0, normal; 1, slow, unprovoked movement; 2, slow, provoked movement; 3, minimal response/unresponsive or spinning), and dehydration (0, normal; 1, ≤ 2 second skin tent, mildly sunken eyes; 2, 2-3 second skin tent, sunken eyes; 3, > 3 second skin tent, sunken eyes). Mice were euthanized if they lost >25% of their starting body weight or became moribund based on clinical scores (a score of 3 in any single category or a cumulative score of ≥9 in respiratory effort, dehydration, temperature reduction, behavior, body condition/appearance).

### Harvest and Processing of Lungs and Blood

Mice were euthanized by 33% isoflurane inhalation. Lungs were aseptically harvested, washed in PBS, and fixed in 10% neutral buffered formalin for histology or digested with collagenase (1 mg/ml, Sigma C0130) and physical homogenization against a 40 µm cell strainer for immune cell staining. Lung digest supernatants were used to quantify cytokines and chemokines and to determine viral and bacterial titers as above; bacterial titers were also measured in peripheral blood. Following red blood cell lysis, lung cells were washed in staining buffer (PBS, 5mM EDTA, 10mM HEPES, and 0.5% bovine serum albumin), counted with trypan blue exclusion using a Cell Countess System (Invitrogen, Grand Island, NY), and prepared for flow cytometric analysis as described below.

### Flow Cytometric Analysis

Flow cytometry (BD FACSAria; San Jose, CA) was performed on single cell suspensions after Fc receptor blocking (TruStainFcX, Biolegend) and viability staining (Zombie Violet Fixable Viability, Biolegend), 25 min surface staining, and fixation (BD Cytofix). The followed anti-mouse antibody panels were used for cell subset analysis: CD45 (clone 30-F11, Pe-Cy7, Biolegend), CD3e (clone 145-2C11, FITC, Biolegend), CD4 (clone RM4-5, V500, BD Biosciences), CD8α (clone 53-6.7, PerCP-Cy5.5, Biolegend), CD19 (clone 6D5, PE, Biolegend), CD335 (clone 29A1.4, APC-Fire750, Biolegend), and CD69 (clone H1.2F3, APC, Biolegend) or CD45 (clone 30-F11, Pe-Cy7, Biolegend), Ly6G (clone 1A8, PerCP-Cy5.5, Biolegend), F4/80 (clone BM8, PE, eBioscience), CD11b (clone M1/70, V500, BD Biosciences), CD11c (clone N418, APC-Fire750, Biolegend), MHC-II (clone I-A/I-E, FITC, eBioscience), and CD69 (clone H1.2F3, APC, Biolegend). The data were analyzed using FlowJo 10.7.2 (Tree Star, Ashland, OR). Data were cleaned using the flowAI application (159) followed by gating viable cells from a forward scatter/side scatter plot, singlet inclusion, and viability dye exclusion. CD45^+^ cells were selected for further analyses. Neutrophils (Ly6G^hi^), alveolar macrophages (AMΦ) (F4/80^hi^CD11c^hi^CD11b^-^MHC-II^low/-^), inflammatory/exudate macrophages (iMΦ) (F4/80^hi^CD11c^hi^CD11b^+^MHC-II^mid/hi^), other monocyte/macrophage populations (F4/80^mid^CD11c^mid^CD11b^+^ and F4/80^mid^CD11c^-^CD11b^+/-^), NK cells (CD3e^-^CD19^-^CD335^+^), CD4 T cells (CD3^+^CD8^-^CD4^+^CD335^-^), CD8 T cells (CD3^+^CD8^+^CD4^-^CD335^-^), NK T cells (CD3e^+^CD335^+^), B cells (CD3e^-^CD19^+^), and recently activated subsets thereof (CD69^+^) were gated as in **Figure S2**.

### Cytokine and Chemokine Quantification

Cytokines G-CSF (CSF-3), GM-CSF, IFN-γ, IL-1α, IL-1β, IL-2, IL-3, IL-4, IL-5, IL-6, IL-9, IL-10, IL-12p70, IL-13, IL-15/IL-15R, IL-17A (CTLA-8), IL-18, IL-22, IL-23, IL-27, IL-28, IL-31, LIF, MCP-3 (CCL7), M-CSF, TNF-α) and chemokines (ENA-78 (CXCL5), eotaxin (CCL11), GROα (CXCL1), IP-10 (CXCL10), MCP-1 (CCL2), MIP-1α (CCL3), MIP-1β (CCL4), MIP-2α (CXCL2), RANTES (CCL5) were measured in lung supernatant by Luminex and ELISA (IFN-α,β). Before use, cell debris and aggregates were removed by centrifugation at 4°C, 400 x *g*. ProcartaPlex magnetic bead cytokine/chemokine plates (Invitrogen) were prepared according to the manufacturer’s instructions. Data were acquired using a MagPix (Luminex) with Luminex xPonent software (v4.2) and analyzed with the ProcartaPlex Analysis App (ThermoFisher Connect). ELISAs for IFNα and IFNβ (PBL Assay Science) were prepared according to the manufacturer’s instructions, read at 450 nm, and analyzed using GraphPad Prism 9.2.0. Mean concentrations of duplicate samples were calculated by the construction of standard curves using a weighted 5PL and 4PL regression for the ProcartaPlex and ELISA data, respectively. Absolute quantities of each cytokine/chemokine were calculated based on the mean concentration of replicate samples normalized to the lung supernatant volume collected during tissue processing. Internal plate controls were used to adjust values obtained between plates and fold changes in cytokine and chemokine quantities were calculated for each animal, normalized to the average of naïve controls (pooled males/females).

### Histology

Following euthanasia and tissue removal as above, lungs were continually fixed in 10% neutral-buffered formalin solution (NBF; ThermoFisher Scientific, Waltham, MA) before being embedded in paraffin, sectioned at 4μm, and mounted on positively charged glass slides (Superfrost Plus; Thermo Fisher Scientific, Waltham, MA). Tissue sections were stained with hematoxylin and eosin (H&E) or subjected to immunohistochemical (IHC) staining to detect SARS-CoV-2 antigen or pneumococcus. Tissue sections were deparaffinized and rehydrated before undergoing antigen retrieval in a citrate-based solution (pH 6.0) at 97°C for SARS-CoV-2 detection or a tris-based solution (pH 9.0) for pneumococcal detection (Vector Laboratories, Burlingame, CA). For IHC, a primary monoclonal antibody against SARS-CoV-2 nucleoprotein (NP) (Sino Biological, Wayne, PA) or a rabbit polyclonal antibody against pneumococcus (Novus Biologicals, Littleton, CO) was used at 1:1000 followed by a biotinylated anti-rabbit antibody (Vector Laboratories, Burlingame, CA) at 1:200, the Vectastain Elite ABC-HRP kit (Vector Laboratories, Burlingame, CA), and 3,3’-Diaminobenzidine (DAB) solution development. Stained sections were counterstained with hematoxylin, dehydrated, and examined by a pathologist blinded to the experimental group assignments. Pathology was scored on a scale from 0-5, where 0 = normal, no tissue affected; 1 = minimal: rare or inconspicuous lesions; 2 = mild: multifocal or small, focal, or widely separated, but conspicuous lesions; 3 = moderate: multifocal, prominent lesions; 4 = marked: extensive to coalescing lesions or areas of inflammation with some loss of structure; 5 = severe: diffuse lesion with effacement of normal structure. Intermediate severity grades were assigned where necessary. To quantify the extent of viral infection in the lungs, digital images of whole lung sections stained for viral antigen were first captured using the Aperio ScanScope XT Slide Scanner (Aperio Technologies, Inc., Vista, CA). The areas of both the entire lung parenchyma (alveoli and bronchioles) and the virus-positive regions were outlined manually with areas determined using ImageScope software (Aperio Technologies, Inc.). Representative images and quantitative analyses of viral spread and lung pathology during infection are shown in **Figures 2**, **5**, respectively.

### Statistical Analysis

Significant differences in Kaplan-Meier survival curves were calculated using the log-rank test. Linear values of lung and blood bacterial loads, viral loads, immune cells, and cytokines/chemokines were compared using an unpaired *t* test with Welch correction except where the Mann-Whitney test was used due to unequal variances (GraphPad Prism 9.2.0 and Rv4.0.3). The confidence interval of significance was set to 95%, and *P* ≤ 0.05 was considered significant.

## Data Availability Statement

The original contributions presented in the study are included in the article/**Supplementary Material**. Further inquiries can be directed to the corresponding authors. The following reagent was deposited by the Centers for Disease Control and Prevention and obtained through BEI Resources, NIAID, NIH: SARS-Related Coronavirus 2, Isolate USA-WA1/2020, NR-52281.

## Ethics Statement

The animal study was reviewed and approved by Institutional Animal Care and Use Committee at the University of Tennessee Health Science Center.

## Author Contributions

APS, RC, CJ, and AMS conceived and designed the experiments. APS, EW, TP, MS, LL, LZ, and YX performed the experiments. YX and PV performed histological analysis. APS and AMS wrote the manuscript with input from all authors. All authors contributed to the article and approved the submitted version.

## Funding

This work was supported by the UTHSC Institute for the Study of Host Pathogen Systems, the University of Tennessee Research Foundation, and NIH grant number AI139088.

## Conflict of Interest

The authors declare that the research was conducted in the absence of any commercial or financial relationships that could be construed as a potential conflict of interest.

## Publisher’s Note

All claims expressed in this article are solely those of the authors and do not necessarily represent those of their affiliated organizations, or those of the publisher, the editors and the reviewers. Any product that may be evaluated in this article, or claim that may be made by its manufacturer, is not guaranteed or endorsed by the publisher.

## References

[1] Toombs JM Van den Abbeele K Democratis J Mandal AKJ Missouris CG. Pneumococcal Coinfection in COVID-19 Patients. J Med Virol (2021) 93:177–9. doi: 10.1002/jmv.26278 PMC7361306 32639585

[2] Tsukamoto T Nakajima N Sakurai A Nakajima M Sakurai E Sato Y. Lung Pathology of Mutually Exclusive Co-Infection With SARS-CoV-2 and Streptococcus Pneumoniae. Emerg Infect Dis (2021) 27:919–23. doi: 10.3201/eid2703.204024 PMC7920656 33443011

[3] Adler H Ball R Fisher M Mortimer K Vardhan MS. Low Rate of Bacterial Co-Infection in Patients With COVID-19. Lancet Microbe (2020) 1:e62. doi: 10.1016/S2666-5247(20)30036-7 32835331 PMC7279742

[4] Zhu X Ge Y Wu T Zhao K Chen Y Wu B. Co-Infection With Respiratory Pathogens Among COVID-2019 Cases. Virus Res (2020) 285:198005. doi: 10.1016/j.virusres.2020.198005 32408156 PMC7213959

[5] He F Xia X Nie D Yang H Jiang Y Huo X. Respiratory Bacterial Pathogen Spectrum Among COVID-19 Infected and Non-COVID-19 Virus Infected Pneumonia Patients. Diagn Microbiol Infect Dis (2020) 98:115199. doi: 10.1016/j.diagmicrobio.2020.115199 32979617 PMC7470696

[6] Garcia-Vidal C Sanjuan G Moreno-Garcia E Puerta-Alcalde P Garcia-Pouton N Chumbita M. Incidence of Co-Infections and Superinfections in Hospitalized Patients With COVID-19: A Retrospective Cohort Study. Clin Microbiol Infect (2021) 27:83–8. doi: 10.1016/j.cmi.2020.07.041 PMC7836762 32745596

[7] Kreitmann L Monard C Dauwalder O Simon M Argaud L. Early Bacterial Co-Infection in ARDS Related to COVID-19. Intensive Care Med (2020) 46:1787–9. doi: 10.1007/s00134-020-06165-5 PMC7358293 32661615

[8] Bordi L Nicastri E Scorzolini L Di Caro A Capobianchi MR Castilletti C. Differential Diagnosis of Illness in Patients Under Investigation for the Novel Coronavirus (SARS-CoV-2), Italy, February 2020. Euro Surveill (2020) 25(8):2000170. doi: 10.2807/1560-7917.ES.2020.25.8.2000170 PMC7055037 32127123

[9] Chen N Zhou M Dong X Qu J Gong F Han Y. Epidemiological and Clinical Characteristics of 99 Cases of 2019 Novel Coronavirus Pneumonia in Wuhan, China: A Descriptive Study. Lancet (2020) 395:507–13. doi: 10.1016/S0140-6736(20)30211-7 PMC7135076 32007143

[10] Kim D Quinn J Pinsky B Shah NH Brown I. Rates of Co-Infection Between SARS-CoV-2 and Other Respiratory Pathogens. JAMA (2020) 323:2085–6. doi: 10.1001/jama.2020.6266 PMC7160748 32293646

[11] Nowak MD Sordillo EM Gitman MR Paniz Mondolfi AE. Coinfection in SARS-CoV-2 Infected Patients: Where Are Influenza Virus and Rhinovirus/Enterovirus? J Med Virol (2020) 92:1699–700. doi: 10.1002/jmv.25953 PMC7267652 32352574

[12] Zhou S Yang Y Zhang X Li Z Liu X Hu C. Clinical Course of 195 Critically Ill COVID-19 Patients: A Retrospective Multicenter Study. Shock (2020) 54:644–51. doi: 10.1097/SHK.0000000000001629 32826818

[13] Hazra A Collison M Pisano J Kumar M Oehler C Ridgway JP. Coinfections With SARS-CoV-2 and Other Respiratory Pathogens. Infect Control Hosp Epidemiol (2020) 41:1228–9. doi: 10.1017/ice.2020.322 PMC7360954 32616098

[14] Koehler P Cornely OA Bottiger BW Dusse F Eichenauer DA Fuchs F. COVID-19 Associated Pulmonary Aspergillosis. Mycoses (2020) 63:528–34. doi: 10.1111/myc.13096 PMC7267243 32339350

[15] Hughes S Troise O Donaldson H Mughal N Moore LSP. Bacterial and Fungal Coinfection Among Hospitalized Patients With COVID-19: A Retrospective Cohort Study in a UK Secondary-Care Setting. Clin Microbiol Infect (2020) 26:1395–9. doi: 10.1016/j.cmi.2020.06.025 PMC7320692 32603803

[16] Massey BW Jayathilake K Meltzer HY. Respiratory Microbial Co-Infection With SARS-CoV-2. Front Microbiol (2020) 11:2079. doi: 10.3389/fmicb.2020.02079 32983056 PMC7477285

[17] Lai CC Wang CY Hsueh PR. Co-Infections Among Patients With COVID-19: The Need for Combination Therapy With Non-Anti-SARS-CoV-2 Agents? J Microbiol Immunol Infect (2020) 53:505–12. doi: 10.1016/j.jmii.2020.05.013 PMC7245213 32482366

[18] Langford BJ So M Raybardhan S Leung V Westwood D MacFadden DR. Bacterial Co-Infection and Secondary Infection in Patients With COVID-19: A Living Rapid Review and Meta-Analysis. Clin Microbiol Infect (2020) 26:1622–9. doi: 10.1016/j.cmi.2020.07.016 PMC7832079 32711058

[19] Richardson S Hirsch JS Narasimhan M Crawford JM McGinn T Davidson KW. Presenting Characteristics, Comorbidities, and Outcomes Among 5700 Patients Hospitalized With COVID-19 in the New York City Area. JAMA (2020) 323:2052–9. doi: 10.1001/jama.2020.6775 PMC7177629 32320003

[20] Singh V Upadhyay P Reddy J Granger J. SARS-CoV-2 Respiratory Co-Infections: Incidence of Viral and Bacterial Co-Pathogens. Int J Infect Dis (2021) 105:617–20. doi: 10.1016/j.ijid.2021.02.087 PMC7905386 33640570

[21] Guan Z Chen C Li Y Yan D Zhang X Jiang D. Impact of Coinfection With SARS-CoV-2 and Influenza on Disease Severity: A Systematic Review and Meta-Analysis. Front Public Health (2021) 9:773130. doi: 10.3389/fpubh.2021.773130 34957025 PMC8703010

[22] Amin-Chowdhury Z Aiano F Mensah A Sheppard C Litt D Fry NK. Impact of the COVID-19 Pandemic on Invasive Pneumococcal Disease and Risk of Pneumococcal Coinfection With SARS-CoV-2: Prospective National Cohort Study, England. Clin Infect Dis (2020) 72(5):e65–75. doi: 10.1093/cid/ciaa1728 PMC7717180 33196783

[23] Shafran N Shafran I Ben-Zvi H Sofer S Sheena L Krause I. Secondary Bacterial Infection in COVID-19 Patients is a Stronger Predictor for Death Compared to Influenza Patients. Sci Rep (2021) 11:12703. doi: 10.1038/s41598-021-92220-0 34135459 PMC8209102

[24] Contou D Claudinon A Pajot O Micaelo M Longuet Flandre P Dubert M. Bacterial and Viral Co-Infections in Patients With Severe SARS-CoV-2 Pneumonia Admitted to a French ICU. Ann Intensive Care (2020) 10:119. doi: 10.1186/s13613-020-00736-x 32894364 PMC7475952

[25] Sharifipour E Shams S Esmkhani M Khodadadi J Fotouhi-Ardakani R Koohpaei A. Evaluation of Bacterial Co-Infections of the Respiratory Tract in COVID-19 Patients Admitted to ICU. BMC Infect Dis (2020) 20:646. doi: 10.1186/s12879-020-05374-z 32873235 PMC7461753

[26] Mirzaei R Goodarzi P Asadi M Soltani A Aljanabi HAA Jeda AS. Bacterial Co-Infections With SARS-CoV-2. IUBMB Life (2020) 72:2097–111. doi: 10.1002/iub.2356 PMC7436231 32770825

[27] Musuuza JS Watson L Parmasad V Putman-Buehler N Christensen L Safdar N. Prevalence and Outcomes of Co-Infection and Superinfection With SARS-CoV-2 and Other Pathogens: A Systematic Review and Meta-Analysis. PloS One (2021) 16:e0251170. doi: 10.1371/journal.pone.0251170 33956882 PMC8101968

[28] Rodriguez-Nava G Yanez-Bello MA Trelles-Garcia DP Chung CW Egoryan G Friedman HJ. A Retrospective Study of Coinfection of SARS-CoV-2 and Streptococcus Pneumoniae in 11 Hospitalized Patients With Severe COVID-19 Pneumonia at a Single Center. Med Sci Monit (2020) 26:e928754. doi: 10.12659/MSM.928754 33188161 PMC7673066

[29] Feldman C Anderson R. The Role of Co-Infections and Secondary Infections in Patients With COVID-19. Pneumonia (Nathan) (2021) 13:5. doi: 10.1186/s41479-021-00083-w 33894790 PMC8068564

[30] Rawson TM Moore LSP Zhu N Ranganathan N Skolimowska K Gilchrist M. Bacterial and Fungal Coinfection in Individuals With Coronavirus: A Rapid Review To Support COVID-19 Antimicrobial Prescribing. Clin Infect Dis (2020) 71:2459–68. doi: 10.1093/cid/ciaa530 PMC7197596 32358954

[31] Bengoechea JA Bamford CG. SARS-CoV-2, Bacterial Co-Infections, and AMR: The Deadly Trio in COVID-19? EMBO Mol Med (2020) 12:e12560. doi: 10.15252/emmm.202012560 32453917 PMC7283846

[32] Bartlett JG Mundy LM. Community-Acquired Pneumonia. N Engl J Med. (1995) 333(24):1618–24. doi: 10.1056/NEJM199512143332408 7477199

[33] Ruiz-Gonzalez A Falguera M Nogues A Rubio-Caballero M. Is Streptococcus Pneumoniae the Leading Cause of Pneumonia of Unknown Etiology? A Microbiologic Study of Lung Aspirates in Consecutive Patients With Community-Acquired Pneumonia. Am J Med (1999) 106:385–90. doi: 10.1016/s0002-9343(99)00050-9 10225239

[34] Bartlett JG Dowell SF Mandell LA File Jr. TM Musher DM Fine MJ. Practice Guidelines for the Management of Community-Acquired Pneumonia in Adults. Infectious Diseases Society of America. Clin Infect Dis (2000) 31:347–82. doi: 10.1086/313954 PMC7109923 10987697

[35] Brundage JF. Interactions Between Influenza and Bacterial Respiratory Pathogens: Implications for Pandemic Preparedness. Lancet Infect Dis (2006) 6:303–12. doi: 10.1016/S1473-3099(06)70466-2 PMC7106411 16631551

[36] McCullers JA. The Co-Pathogenesis of Influenza Viruses With Bacteria in the Lung. Nat Rev Microbiol (2014) 12:252–62. doi: 10.1038/nrmicro3231 24590244

[37] Morris DE Cleary DW Clarke SC. Secondary Bacterial Infections Associated With Influenza Pandemics. Front Microbiol (2017) 8:1041. doi: 10.3389/fmicb.2017.01041 28690590 PMC5481322

[38] Cawcutt K Kalil AC. Pneumonia With Bacterial and Viral Coinfection. Curr Opin Crit Care (2017) 23:385–90. doi: 10.1097/MCC.0000000000000435 28777158

[39] Smith AM McCullers JA. Secondary Bacterial Infections in Influenza Virus Infection Pathogenesis. In: Compans R Oldstone M (eds) Influenza Pathogenesis and Control - Volume I. Current Topics in Microbiology and Immunology, vol 385. Cham: Springer (2014). doi: 10.1007/82_2014_394 PMC7122299 25027822

[40] Short KR Habets MN Hermans PWM Diavatopoulos DA. Interactions Between Streptococcus Pneumoniae and Influenza Virus: A Mutually Beneficial Relationship? Future Microbiol (2012) 7:609–24. doi: 10.2217/fmb.12.29 22568716

[41] Metzger DW Sun K. Immune Dysfunction and Bacterial Coinfections Following Influenza. J Immunol (2013) 191:2047–52. doi: 10.4049/jimmunol.1301152 PMC3760235 23964104

[42] Smith AM. Host-Pathogen Kinetics During Influenza Infection and Coinfection: Insights From Predictive Modeling. Immunol Rev (2018) 285:97–112. doi: 10.1111/imr.12692 30129197 PMC6175135

[43] Weinberger DM Simonsen L Jordan R Steiner C Miller M Viboud C. Impact of the 2009 Influenza Pandemic on Pneumococcal Pneumonia Hospitalizations in the United States. J Infect Dis (2012) 205:458–65. doi: 10.1093/infdis/jir749 PMC3276240 22158564

[44] Robinson KM Kolls JK Alcorn JF. The Immunology of Influenza Virus-Associated Bacterial Pneumonia. Curr Opin Immunol (2015) 34:59–67. doi: 10.1016/j.coi.2015.02.002 25723597 PMC4444379

[45] Rynda-Apple A Robinson KM Alcorn JF. Influenza and Bacterial Superinfection: Illuminating the Immunologic Mechanisms of Disease. Infect Immun (2015) 83:3764–70. doi: 10.1128/IAI.00298-15 PMC4567631 26216421

[46] Bellinghausen C Rohde GGU Savelkoul PHM Wouters EFM Stassen FRM. Viral-Bacterial Interactions in the Respiratory Tract. J Gen Virol (2016) 97:3089–102. doi: 10.1099/jgv.0.000627 27902340

[47] Chien Y-W Klugman KP Morens DM. Bacterial Pathogens and Death During the 1918 Influenza Pandemic. N Engl J Med (2009) 361:2582–3. doi: 10.1056/NEJMc0908216 20032332

[48] MacIntyre CR Chughtai AA Barnes M Ridda I Seale H Toms R. The Role of Pneumonia and Secondary Bacterial Infection in Fatal and Serious Outcomes of Pandemic Influenza a(H1N1)Pdm09. BMC Infect Dis (2018) 18:637. doi: 10.1186/s12879-018-3548-0 30526505 PMC6286525

[49] Gill JR Sheng Z Ely SF Guinee DG Beasley MB Suh J. Pulmonary Pathologic Findings of Fatal 2009 Pandemic Influenza A/H1N1 Viral Infections. Arch Pathol Lab Med (2010) 134:235–43. doi: 10.1043/1543-2165-134.2.235 PMC2819217 20121613

[50] Morens David M Taubenberger Jeffery K Fauci AS. Predominant Role of Bacterial Pneumonia as a Cause of Death in Pandemic Influenza: Implications for Pandemic Influenza Preparedness. J Infect Dis (2008) 198:962–70. doi: 10.1086/591708 PMC2599911 18710327

[51] Petersen E Koopmans M Go U Hamer DH Petrosillo N Castelli F. Comparing SARS-CoV-2 With SARS-CoV and Influenza Pandemics. Lancet Infect Dis (2020) 20:e238–44. doi: 10.1016/S1473-3099(20)30484-9 PMC7333991 32628905

[52] Abdelrahman Z Li M Wang X. Comparative Review of SARS-CoV-2, SARS-CoV, MERS-CoV, and Influenza A Respiratory Viruses. Front Immunol (2020) 11:552909. doi: 10.3389/fimmu.2020.552909 33013925 PMC7516028

[53] Simmonds P Williams S Harvala H. Understanding the Outcomes of COVID-19 - Does the Current Model of an Acute Respiratory Infection Really Fit? J Gen Virol (2021) 102(3):001545. doi: 10.1099/jgv.0.001545 PMC8222868 33331810

[54] Flerlage T Boyd DF Meliopoulos V Thomas PG Schultz-Cherry S. Influenza Virus and SARS-CoV-2: Pathogenesis and Host Responses in the Respiratory Tract. Nat Rev Microbiol (2021) 19:425–41. doi: 10.1038/s41579-021-00542-7 PMC8023351 33824495

[55] Smith AP Lane LC Ramirez Zuniga I Moquin D Vogel P Smith AM. Increased Virus Dissemination Leads to Enhanced Lung Injury But Not Inflammation During Influenza-Associated Secondary Bacterial Infection. 10.1093/femsmc/xtac022 PMC10117793 37332507

[56] Myers MA Smith AP Lane LC Moquin DJ Aogo R Woolard S. Dynamically Linking Influenza Virus Infection Kinetics, Lung Injury, Inflammation, and Disease Severity. Elife (2021) 10:e68864. doi: 10.7554/eLife.68864 34282728 PMC8370774

[57] Granados A Peci A McGeer A Gubbay JB. Influenza and Rhinovirus Viral Load and Disease Severity in Upper Respiratory Tract Infections. J Clin Virol (2017) 86:14–9. doi: 10.1016/j.jcv.2016.11.008 27893998

[58] Oshansky CM Gartland AJ Wong SS Jeevan T Wang D Roddam PL. Mucosal Immune Responses Predict Clinical Outcomes During Influenza Infection Independently of Age and Viral Load. Am J Respir Crit Care Med (2014) 189:449–62. doi: 10.1164/rccm.201309-1616OC PMC3977720 24308446

[59] Lee CK Lee HK Loh TP Lai FY Tambyah PA Chiu L. Comparison of Pandemic (H1N1) 2009 and Seasonal Influenza Viral Loads, Singapore. Emerg Infect Dis (2011) 17:287–91. doi: 10.3201/eid1702.100282 PMC3204747 21291608

[60] Abdulrahman A Mallah SI Alqahtani M. COVID-19 Viral Load Not Associated With Disease Severity: Findings From a Retrospective Cohort Study. BMC Infect Dis (2021) 21:688. doi: 10.1186/s12879-021-06376-1 34271860 PMC8284033

[61] Cocconcelli E Castelli G Onelia F Lavezzo E Giraudo C Bernardinello N. Disease Severity and Prognosis of SARS-CoV-2 Infection in Hospitalized Patients Is Not Associated With Viral Load in Nasopharyngeal Swab. Front Med (Lausanne) (2021) 8:714221. doi: 10.3389/fmed.2021.714221 34568371 PMC8460755

[62] Mudd PA Crawford JC Turner JS Souquette A Reynolds D Bender D. Distinct Inflammatory Profiles Distinguish COVID-19 From Influenza With Limited Contributions From Cytokine Storm. Sci Adv (2020) 6(50):eabe3024. doi: 10.1126/sciadv.abe3024 33187979 PMC7725462

[63] Paget C Trottein F. COVID-19 and Flu: Conserved or Specific Immune Signature? Cell Mol Immunol (2021) 18:245–6. doi: 10.1038/s41423-020-00595-3 PMC7797702 33432063

[64] Zhu L Yang P Zhao Y Zhuang Z Wang Z Song R. Single-Cell Sequencing of Peripheral Mononuclear Cells Reveals Distinct Immune Response Landscapes of COVID-19 and Influenza Patients. Immunity (2020) 53:685–696 e683. doi: 10.1016/j.immuni.2020.07.009 32783921 PMC7368915

[65] Lee JS Park S Jeong HW Ahn JY Choi SJ Lee H. Immunophenotyping of COVID-19 and Influenza Highlights the Role of Type I Interferons in Development of Severe COVID-19. Sci Immunol (2020) 5(49):eabd1554. doi: 10.1126/sciimmunol.abd1554 32651212 PMC7402635

[66] Galani IE Rovina N Lampropoulou V Triantafyllia V Manioudaki M Pavlos E. Untuned Antiviral Immunity in COVID-19 Revealed by Temporal Type I/III Interferon Patterns and Flu Comparison. Nat Immunol (2021) 22:32–40. doi: 10.1038/s41590-020-00840-x 33277638

[67] Ghoneim HE Thomas PG McCullers JA. Depletion of Alveolar Macrophages During Influenza Infection Facilitates Bacterial Superinfections. J Immunol (2013) 191:1250–9. doi: 10.4049/jimmunol.1300014 PMC4907362 23804714

[68] Smith AM Adler FR Ribeiro RM Gutenkunst RN McAuley JL McCullers JA. Kinetics of Coinfection With Influenza A Virus and Streptococcus Pneumoniae. PloS Pathog (2013) 9:e1003238. doi: 10.1371/journal.ppat.1003238 23555251 PMC3605146

[69] Smith AM Smith AP. A Critical, Nonlinear Threshold Dictates Bacterial Invasion and Initial Kinetics During Influenza. Sci Rep (2016) 6(1):38703. doi: 10.1038/srep38703 27974820 PMC5156930

[70] Califano D Furuya Y Metzger DW. Effects of Influenza on Alveolar Macrophage Viability Are Dependent on Mouse Genetic Strain. J Immunol (2018) 201:134–44. doi: 10.4049/jimmunol.1701406 PMC6008236 29760191

[71] Shahangian A Chow EK Tian X Kang JR Ghaffari A Liu SY. Type I IFNs Mediate Development of Postinfluenza Bacterial Pneumonia in Mice. J Clin Invest (2009) 119:1910–20. doi: 10.1172/JCI35412 PMC2701856 19487810

[72] McNamee LA Harmsen AG. Both Influenza-Induced Neutrophil Dysfunction and Neutrophil-Independent Mechanisms Contribute to Increased Susceptibility to a Secondary Streptococcus Pneumoniae Infection. Infect Immun (2006) 74:6707–21. doi: 10.1128/IAI.00789-06 PMC1698099 16982840

[73] Knoll R Schultze JL Schulte-Schrepping J. Monocytes and Macrophages in COVID-19. Front Immunol (2021) 12:720109. doi: 10.3389/fimmu.2021.720109 34367190 PMC8335157

[74] Reusch N De Domenico E Bonaguro L Schulte-Schrepping J Baßler K Schultze JL. Neutrophils in COVID-19. Front Immunol (2021) 12:652470. doi: 10.3389/fimmu.2021.652470 33841435 PMC8027077

[75] Schulte-Schrepping J Reusch N Paclik D Baßler K Schlickeiser S Zhang B. Severe COVID-19 Is Marked by a Dysregulated Myeloid Cell Compartment. Cell (2020) 182:1419–1440 e1423. doi: 10.1016/j.cell.2020.08.001 32810438 PMC7405822

[76] Ami Y Nagata N Shirato K Watanabe R Iwata N Nakagaki K. Co-Infection of Respiratory Bacterium With Severe Acute Respiratory Syndrome Coronavirus Induces an Exacerbated Pneumonia in Mice. Microbiol Immunol (2008) 52:118–27. doi: 10.1111/j.1348-0421.2008.00011.x PMC7168413 18380809

[77] McCullers JA Rehg JE. Lethal Synergism Between Influenza Virus and Streptococcus Pneumoniae: Characterization of a Mouse Model and the Role of Platelet-Activating Factor Receptor. J Infect Dis (2002) 186:341–50. doi: 10.1086/341462 12134230

[78] Abramson JS Lewis JC Lyles DS Heller KA Mills EL Bass DA. Inhibition of Neutrophil Lysosome-Phagosome Fusion Associated With Influenza Virus Infection *In Vitro.* Role in Depressed Bactericidal Activity. J Clin Invest (1982) 69:1393–7. doi: 10.1172/JCI110580 PMC370213 7085879

[79] Abramson JS Wheeler JG. Virus-Induced Neutrophil Dysfunction: Role in the Pathogenesis of Bacterial Infections. Pediatr Infect Dis J (1994) 13:643–52. doi: 10.1097/00006454-199407000-00012 7970955

[80] Colamussi ML White MR Crouch E Hartshorn KL. Influenza A Virus Accelerates Neutrophil Apoptosis and Markedly Potentiates Apoptotic Effects of Bacteria. Blood (1999) 93:2395–403. doi: 10.1182/blood.V93.7.2395 10090951

[81] Engelich G White M Hartshorn KL. Neutrophil Survival Is Markedly Reduced by Incubation With Influenza Virus and Streptococcus Pneumoniae: Role of Respiratory Burst. J Leukoc Biol (2001) 69:50–6. doi: 10.1189/jlb.69.1.50 11200067

[82] Smith AP Lane LC van Opijnen T Woolard S Carter R Iverson A. Dynamic Pneumococcal Genetic Adaptations Support Bacterial Growth and Inflammation During Coinfection With Influenza. Infect Immun (2021) 89:e0002321. doi: 10.1128/IAI.00023-21 33875471 PMC8208518

[83] Shepardson KM Larson K Morton RV Prigge JR Schmidt EE Huber VC. Differential Type I Interferon Signaling Is a Master Regulator of Susceptibility to Postinfluenza Bacterial Superinfection. MBio (2016) 7(3):e00506-16. doi: 10.1128/mBio.00506-16 27143388 PMC4959663

[84] McCullers JA. Insights Into the Interaction Between Influenza Virus and Pneumococcus. Clin Microbiol Rev (2006) 19:571–82. doi: 10.1128/CMR.00058-05 PMC1539103 16847087

[85] Kash JC Taubenberger JK. The Role of Viral, Host, and Secondary Bacterial Factors in Influenza Pathogenesis. Am J Pathol (2015) 185:1528–36. doi: 10.1016/j.ajpath.2014.08.030 PMC4450310 25747532

[86] Chu H Chan JF Wang Y Yuen TT Chai Y Hou Y. Comparative Replication and Immune Activation Profiles of SARS-CoV-2 and SARS-CoV in Human Lungs: An *Ex Vivo* Study With Implications for the Pathogenesis of COVID-19. Clin Infect Dis (2020) 71:1400–9. doi: 10.1093/cid/ciaa410 PMC7184390 32270184

[87] Lv J Wang Z Qu Y Zhu H Zhu Q Tong W. Distinct Uptake, Amplification, and Release of SARS-CoV-2 by M1 and M2 Alveolar Macrophages. Cell Discov (2021) 7:24. doi: 10.1038/s41421-021-00258-1 33850112 PMC8043100

[88] Dalskov L Møhlenberg M Thyrsted J Blay-Cadanet J Poulsen ET Folkersen BH. SARS-CoV-2 Evades Immune Detection in Alveolar Macrophages. EMBO Rep (2020) 21:e51252. doi: 10.15252/embr.202051252 33112036 PMC7645910

[89] Grant RA Morales-Nebreda L Markov NS Swaminathan S Querrey M Guzman ER. Circuits Between Infected Macrophages and T Cells in SARS-CoV-2 Pneumonia. Nature (2021) 590:635–41. doi: 10.1038/s41586-020-03148-w PMC7987233 33429418

[90] Taefehshokr N Taefehshokr S Hemmat N Heit B. Covid-19: Perspectives on Innate Immune Evasion. Front Immunol (2020) 11:580641. doi: 10.3389/fimmu.2020.580641 33101306 PMC7554241

[91] Beyer DK Forero A. Mechanisms of Antiviral Immune Evasion of SARS-CoV-2. J Mol Biol (2021) 167265. doi: 10.1016/j.jmb.2021.167265 PMC8457632 34562466

[92] Lazarevic I Pravica V Miljanovic D Cupic M. Immune Evasion of SARS-CoV-2 Emerging Variants: What Have We Learnt So Far? Viruses (2021) 13(7):1192. doi: 10.3390/v13071192 34206453 PMC8310325

[93] Sette A Crotty S. Adaptive Immunity to SARS-CoV-2 and COVID-19. Cell (2021) 184:861–80. doi: 10.1016/j.cell.2021.01.007 PMC7803150 33497610

[94] Schmolke M Garcia-Sastre A. Evasion of Innate and Adaptive Immune Responses by Influenza A Virus. Cell Microbiol (2010) 12:873–80. doi: 10.1111/j.1462-5822.2010.01475.x PMC2897956 20482552

[95] Kikkert M. Innate Immune Evasion by Human Respiratory RNA Viruses. J Innate Immun (2020) 12:4–20. doi: 10.1159/000503030 31610541 PMC6959104

[96] Blevins LK Wren JT Holbrook BC Hayward SL Swords WE Parks GD. Coinfection With Streptococcus Pneumoniae Negatively Modulates the Size and Composition of the Ongoing Influenza-Specific CD8 + T Cell Response. J Immunol (2014) 193:5076–87. doi: 10.4049/jimmunol.1400529 PMC4265766 25311807

[97] Rowe HM Meliopoulos VA Iverson A Bomme P Schultz-Cherry S Rosch JW. Direct Interactions With Influenza Promote Bacterial Adherence During Respiratory Infections. Nat Microbiol (2019) 4:1328–36. doi: 10.1038/s41564-019-0447-0 PMC7069060 31110359

[98] Warnking K Klemm C Löffler B Niemann S van Krüchten A Peters G. Super-Infection With S Taphylococcus Aureus Inhibits Influenza Virus-Induced Type I IFN Signalling Through Impaired STAT1-STAT2 Dimerization: Influenza Virus- and S. Aureus -Mediated Signalling. Cell Microbiol (2015) 17:303–17. doi: 10.1111/cmi.12375 25293394

[99] Mathew D Giles JR Baxter AE Oldridge DA Greenplate AR Wu JE. Deep Immune Profiling of COVID-19 Patients Reveals Distinct Immunotypes With Therapeutic Implications. Science (2020) 369:1210. doi: 10.1126/science.abc8511 32669297 PMC7402624

[100] Song JW Zhang C Fan X Meng P Xu Z Xia P. Immunological and Inflammatory Profiles in Mild and Severe Cases of COVID-19. Nat Commun (2020) 11:3410. doi: 10.1038/s41467-020-17240-2 32641700 PMC7343781

[101] Kuri-Cervantes L Pampena MB Meng W Rosenfeld AM Ittner CAG Weisman AR. Comprehensive Mapping of Immune Perturbations Associated With Severe COVID-19. Sci Immunol (2020) 5:eabd7114. doi: 10.1126/sciimmunol.abd7114 32669287 PMC7402634

[102] Zheng HY Zhang M Yang CX Zhang N Wang XC Yang XP. Elevated Exhaustion Levels and Reduced Functional Diversity of T Cells in Peripheral Blood May Predict Severe Progression in COVID-19 Patients. Cell Mol Immunol (2020) 17:541–3. doi: 10.1038/s41423-020-0401-3 PMC7091621 32203186

[103] Diao B Wang C Tan Y Chen X Liu Y Ning L. Reduction and Functional Exhaustion of T Cells in Patients With Coronavirus Disease 2019 (COVID-19). Front Immunol (2020) 11:827. doi: 10.3389/fimmu.2020.00827 32425950 PMC7205903

[104] Brueggemann AB Jansen van Rensburg MJ Shaw D McCarthy ND Jolley KA Maiden MCJ. Changes in the Incidence of Invasive Disease Due to Streptococcus Pneumoniae, Haemophilus Influenzae, and Neisseria Meningitidis During the COVID-19 Pandemic in 26 Countries and Territories in the Invasive Respiratory Infection Surveillance Initiative: A Prospective Analysis of Surveillance Data. Lancet Digit Health (2021) 3:e360–70. doi: 10.1016/S2589-7500(21)00077-7 PMC8166576 34045002

[105] Huh K Kim Y Ji W Kim DW Lee E Kim J. Decrease in Hospital Admissions for Respiratory Diseases During the COVID-19 Pandemic: A Nationwide Claims Study. Thorax (2021) 76:939–41. doi: 10.1136/thoraxjnl-2020-216526 33782081

[106] Rodgers L Sheppard M Smith A Dietz S Jayanthi P Yuan Y Bull L. Changes in Seasonal Respiratory Illnesses in the United States During the Coronavirus Disease 2019 (COVID-19) Pandemic. Clin Infect Dis (2021) 73:S110–7. doi: 10.1093/cid/ciab311 PMC8135472 33912902

[107] Olsen SJ Winn AK Budd AP Prill MM Steel J Midgley CM. Changes in Influenza and Other Respiratory Virus Activity During the COVID-19 Pandemic-United States, 2020-2021. Am J Transplant (2021) 21:3481–6. doi: 10.1111/ajt.16049 PMC8653380 34624182

[108] Huang QS Wood T Jelley L Jennings T Jefferies S Daniells K. Impact of the COVID-19 Nonpharmaceutical Interventions on Influenza and Other Respiratory Viral Infections in New Zealand. Nat Commun (2021) 12:1001. doi: 10.1038/s41467-021-21157-9 33579926 PMC7881137

[109] Antonelli M Penfold R Merino J Sudre C Molteni E Berry S. Risk Factors and Disease Profile of Post-Vaccination SARS-CoV-2 Infection in UK Users of the COVID Symptom Study App: A Prospective, Community-Based, Nested, Case-Control Study. Lancet Infect Dis (2021) 22(1):43–55. doi: 10.1016/S1473-3099(21)00460-6 34480857 PMC8409907

[110] Tenforde MW Self WH Adams K Gaglani M Ginde AA McNeal T. Association Between mRNA Vaccination and COVID-19 Hospitalization and Disease Severity. JAMA (2021) 326(20):2043–54. doi: 10.1001/jama.2021.19499 PMC8569602 34734975

[111] National Center for Immunization and Respiratory Diseases (NCIRD), D. o. V. D. The Possibility of COVID-19 After Vaccination: Breakthrough Infections (2021). Available at: https://www.cdc.gov/coronavirus/2019-ncov/vaccines/effectiveness/why-measure-effectiveness/breakthrough-cases.html.

[112] Hament JM Kimpen JL Fleer A Wolfs TF. Respiratory Viral Infection Predisposing for Bacterial Disease: A Concise Review. FEMS Immunol Med Microbiol (1999) 26:189–95. doi: 10.1111/j.1574-695X.1999.tb01389.x 10575129

[113] Karlström Å Heston SM Boyd KL Tuomanen EI McCullers JA. Toll-Like Receptor 2 Mediates Fatal Immunopathology in Mice During Treatment of Secondary Pneumococcal Pneumonia Following Influenza. J Infect Dis (2011) 204:1358–66. doi: 10.1093/infdis/jir522 PMC3218647 21900488

[114] LeVine AM Koeningsknecht V Stark JM. Decreased Pulmonary Clearance of S. Pneumoniae Following Influenza A Infection in Mice. J Virol Methods (2001) 94:173–86. doi: 10.1016/S0166-0934(01)00287-7 11337052

[115] Verma AK Bansal S Bauer C Muralidharan A Sun K. Influenza Infection Induces Alveolar Macrophage Dysfunction and Thereby Enables Noninvasive Streptococcus Pneumoniae to Cause Deadly Pneumonia. J Immunol (2020) 205:1601–7. doi: 10.4049/jimmunol.2000094 PMC7484308 32796026

[116] Hang do TT Choi EJ Song JY Kim SE Kwak J Shin YK. Differential Effect of Prior Influenza Infection on Alveolar Macrophage Phagocytosis of Staphylococcus Aureus and Escherichia Coli: Involvement of Interferon-Gamma Production. Microbiol Immunol (2011) 55:751–9. doi: 10.1111/j.1348-0421.2011.00383.x 21895747

[117] Shibata T Makino A Ogata R Nakamura S Ito T Nagata K. Respiratory Syncytial Virus Infection Exacerbates Pneumococcal Pneumonia *via* Gas6/Axl-Mediated Macrophage Polarization. J Clin Invest (2020) 130:3021–37. doi: 10.1172/JCI125505 PMC7260035 32364537

[118] McCullers JA Bartmess KC. Role of Neuraminidase in Lethal Synergism Between Influenza Virus and Streptococcus Pneumoniae. J Infect Dis (2003) 187:1000–9. doi: 10.1086/368163 12660947

[119] Plotkowski MC Puchelle E Beck G Jacquot J Hannoun C. Adherence of Type I Streptococcus Pneumoniae to Tracheal Epithelium of Mice Infected With Influenza A/PR8 Virus. Am Rev Respir Dis (1986) 134:1040–4. doi: 10.1164/arrd.1986.134.5.1040 3777666

[120] Avadhanula V Rodriguez CA Devincenzo P Wang Y Webby RJ Ulett GC. Respiratory Viruses Augment the Adhesion of Bacterial Pathogens to Respiratory Epithelium in a Viral Species- and Cell Type-Dependent Manner. J Virol (2006) 80:1629–36. doi: 10.1128/JVI.80.4.1629-1636.2006 PMC1367158 16439519

[121] Stark JM Stark MA Colasurdo GN LeVine AM. Decreased Bacterial Clearance From the Lungs of Mice Following Primary Respiratory Syncytial Virus Infection. J Med Virol (2006) 78:829–38. doi: 10.1002/jmv.20631 16628585

[122] Rezaee F DeSando SA Ivanov AI Chapman TJ Knowlden SA Beck LA. Sustained Protein Kinase D Activation Mediates Respiratory Syncytial Virus-Induced Airway Barrier Disruption. J Virol (2013) 87:11088–95. doi: 10.1128/JVI.01573-13 PMC3807305 23926335

[123] Ruan T Sun J Liu W Prinz RA Peng D Liu X Xu X. H1N1 Influenza Virus Cross-Activates Gli1 to Disrupt the Intercellular Junctions of Alveolar Epithelial Cells. Cell Rep (2020) 31:107801. doi: 10.1016/j.celrep.2020.107801 32610119

[124] Shepley-McTaggart A Sagum CA Oliva I Rybakovsky E DiGuilio K Liang J. SARS-CoV-2 Envelope (E) Protein Interacts With PDZ-Domain-2 of Host Tight Junction Protein ZO1. PloS One (2021) 16:e0251955. doi: 10.1371/journal.pone.0251955 34106957 PMC8189464

[125] De Maio F Lo Cascio E Babini G Sali M Della Longa S Tilocca B. Improved Binding of SARS-CoV-2 Envelope Protein to Tight Junction-Associated PALS1 Could Play a Key Role in COVID-19 Pathogenesis. Microbes Infect (2020) 22:592–7. doi: 10.1016/j.micinf.2020.08.006 PMC7473260 32891874

[126] Teoh KT Siu YL Chan WL Schlüter MA Liu CJ Peiris JS. The SARS Coronavirus E Protein Interacts With PALS1 and Alters Tight Junction Formation and Epithelial Morphogenesis. Mol Biol Cell (2010) 21:3838–52. doi: 10.1091/mbc.E10-04-0338 PMC2982091 20861307

[127] Nakamura S Davis KM Weiser JN. Synergistic Stimulation of Type I Interferons During Influenza Virus Coinfection Promotes Streptococcus Pneumoniae Colonization in Mice. J Clin Invest (2011) 121:3657–65. doi: 10.1172/JCI57762 PMC3163966 21841308

[128] Channappanavar R Fehr AR Vijay R Mack M Zhao J Meyerholz DK. Dysregulated Type I Interferon and Inflammatory Monocyte-Macrophage Responses Cause Lethal Pneumonia in SARS-CoV-Infected Mice. Cell Host Microbe (2016) 19:181–93. doi: 10.1016/j.chom.2016.01.007 PMC4752723 26867177

[129] Zhou Z Ren L Zhang L Zhong J Xiao Y Jia Z. Heightened Innate Immune Responses in the Respiratory Tract of COVID-19 Patients. Cell Host Microbe (2020) 27:883–890 e882. doi: 10.1016/j.chom.2020.04.017 32407669 PMC7196896

[130] Blanco-Melo D Nilsson-Payant BE Liu WC Uhl S Hoagland D Møller R. Imbalanced Host Response to SARS-CoV-2 Drives Development of COVID-19. Cell (2020) 181:1036–1045 e1039. doi: 10.1016/j.cell.2020.04.026 32416070 PMC7227586

[131] Weeks-Gorospe JN Hurtig HR Iverson AR Schuneman MJ Webby RJ McCullers JA. Naturally Occurring Swine Influenza A Virus PB1-F2 Phenotypes That Contribute to Superinfection With Gram-Positive Respiratory Pathogens. J Virol (2012) 86:9035–43. doi: 10.1128/JVI.00369-12 PMC3416121 22674997

[132] Iverson AR Boyd KL McAuley JL Plano LR Hart ME McCullers JA. Influenza Virus Primes Mice for Pneumonia From Staphylococcus Aureus. J Infect Dis (2011) 203:880–8. doi: 10.1093/infdis/jiq113 PMC3071123 21278211

[133] Tashiro M Ciborowski P Klenk HD Pulverer G Rott R. Role of Staphylococcus Protease in the Development of Influenza Pneumonia. Nature (1987) 325:536–7. doi: 10.1038/325536a0 3543690

[134] Hament JM Aerts PC Fleer A van Dijk H Harmsen T Kimpen JL. Direct Binding of Respiratory Syncytial Virus to Pneumococci: A Phenomenon That Enhances Both Pneumococcal Adherence to Human Epithelial Cells and Pneumococcal Invasiveness in a Murine Model. Pediatr Res (2005) 58:1198–203. doi: 10.1203/01.pdr.0000188699.55279.1b 16306193

[135] Weiser JN Ferreira DM Paton JC. Streptococcus Pneumoniae: Transmission, Colonization and Invasion. Nat Rev Microbiol (2018) 16:355–67. doi: 10.1038/s41579-018-0001-8 PMC5949087 29599457

[136] Hoffmann J Machado D Terrier O Pouzol S Messaoudi M Basualdo W. Viral and Bacterial Co-Infection in Severe Pneumonia Triggers Innate Immune Responses and Specifically Enhances IP-10: A Translational Study. Sci Rep (2016) 6:38532. doi: 10.1038/srep38532 27922126 PMC5138590

[137] Machado D Hoffmann J Moroso M Rosa-Calatrava M Endtz H Terrier O. RSV Infection in Human Macrophages Promotes CXCL10/IP-10 Expression During Bacterial Co-Infection. Int J Mol Sci (2017) 18:2654. doi: 10.3390/ijms18122654 PMC5751256 29215596

[138] Taghavi S Jackson-Weaver O Abdullah S Wanek A Drury R Packer J. Interleukin-22 Mitigates Acute Respiratory Distress Syndrome (ARDS). PloS One (2021) 16:e0254985. doi: 10.1371/journal.pone.0254985 34597299 PMC8486146

[139] Xu F Liu Q Lin S Shen N Yin Y Cao J. IL-27 Is Elevated in Acute Lung Injury and Mediates Inflammation. J Clin Immunol (2013) 33:1257–68. doi: 10.1007/s10875-013-9923-0 PMC7102048 23842867

[140] Pociask DA Scheller EV Mandalapu S McHugh KJ Enelow RI Fattman CL. IL-22 is Essential for Lung Epithelial Repair Following Influenza Infection. Am J Pathol (2013) 182:1286–96. doi: 10.1016/j.ajpath.2012.12.007 PMC3620404 23490254

[141] Liu FD Kenngott EE Schröter MF Kühl A Jennrich S Watzlawick R. Timed Action of IL-27 Protects From Immunopathology While Preserving Defense in Influenza. PloS Pathog (2014) 10:e1004110. doi: 10.1371/journal.ppat.1004110 24809349 PMC4014457

[142] Ivanov S Renneson J Fontaine J Barthelemy A Paget C Fernandez EM. Interleukin-22 Reduces Lung Inflammation During Influenza A Virus Infection and Protects Against Secondary Bacterial Infection. J Virol (2013) 87:6911–24. doi: 10.1128/JVI.02943-12 PMC3676141 23596287

[143] Barthelemy A Sencio V Soulard D Deruyter L Faveeuw C Le Goffic R. Interleukin-22 Immunotherapy During Severe Influenza Enhances Lung Tissue Integrity and Reduces Secondary Bacterial Systemic Invasion. Infect Immun (2018) 86:e00706-17. doi: 10.1128/IAI.00706-17 29661933 PMC6013680

[144] Weitnauer M Mijosek V Dalpke AH. Control of Local Immunity by Airway Epithelial Cells. Mucosal Immunol (2016) 9:287–98. doi: 10.1038/mi.2015.126 26627458

[145] Atamas SP Chapoval SP Keegan AD. Cytokines in Chronic Respiratory Diseases. F1000 Biol Rep (2013) 5:3. doi: 10.3410/B5-3 23413371 PMC3564216

[146] Gieseck RL 3rd Wilson MS Wynn TA. Type 2 Immunity in Tissue Repair and Fibrosis. Nat Rev Immunol (2018) 18:62–76. doi: 10.1038/nri.2017.90 28853443

[147] Fulkerson PC Fischetti CA Hassman LM Nikolaidis NM Rothenberg ME. Persistent Effects Induced by IL-13 in the Lung. Am J Respir Cell Mol Biol (2006) 35:337–46. doi: 10.1165/rcmb.2005-0474OC PMC2643287 16645178

[148] Marone G Granata F Pucino V Pecoraro A Heffler E Loffredo S. The Intriguing Role of Interleukin 13 in the Pathophysiology of Asthma. Front Pharmacol (2019) 10:1387. doi: 10.3389/fphar.2019.01387 31866859 PMC6908970

[149] Smith AM Huber VC. The Unexpected Impact of Vaccines on Secondary Bacterial Infections Following Influenza. Viral Immunol (2018) 31:159–73. doi: 10.1089/vim.2017.0138 PMC5863092 29148920

[150] Haas EJ Angulo FJ McLaughlin JM Anis E Singer SR Khan F. Impact and Effectiveness of mRNA BNT162b2 Vaccine Against SARS-CoV-2 Infections and COVID-19 Cases, Hospitalisations, and Deaths Following a Nationwide Vaccination Campaign in Israel: An Observational Study Using National Surveillance Data. Lancet (2021) 397:1819–29. doi: 10.1016/S0140-6736(21)00947-8 PMC8099315 33964222

[151] Polack FP Thomas SJ Kitchin N Absalon J Gurtman A Lockhart S. Safety and Efficacy of the BNT162b2 mRNA Covid-19 Vaccine. N Engl J Med (2020) 383:2603–15. doi: 10.1056/NEJMoa2034577 PMC7745181 33301246

[152] Dagan N Barda N Kepten E Miron O Perchik S Katz MA. BNT162b2 mRNA Covid-19 Vaccine in a Nationwide Mass Vaccination Setting. N Engl J Med (2021) 384:1412–23. doi: 10.1056/NEJMoa2101765 PMC7944975 33626250

[153] Baden LR El Sahly HM Essink B Kotloff K Frey S Novak R. Efficacy and Safety of the mRNA-1273 SARS-CoV-2 Vaccine. N Engl J Med (2021) 384:403–16. doi: 10.1056/NEJMoa2035389 PMC7787219 33378609

[154] Bergwerk M Gonen T Lustig Y Amit S Lipsitch M Cohen C. Covid-19 Breakthrough Infections in Vaccinated Health Care Workers. N Engl J Med (2021) 385:1474–84. doi: 10.1056/NEJMoa2109072 PMC8362591 34320281

[155] Lipsitch M Krammer F Regev-Yochay G Lustig Y Balicer RD. SARS-CoV-2 Breakthrough Infections in Vaccinated Individuals: Measurement, Causes and Impact. Nat Rev Immunol (2022) 22:57–65. doi: 10.1038/s41577-021-00662-4 34876702 PMC8649989

[156] Brown C Vostok J Johnson H Burns M Gharpure R Sami S. Outbreak of SARS-CoV-2 Infections, Including COVID-19 Vaccine Breakthrough Infections, Associated With Large Public Gatherings - Barnstable County, Massachusetts, July 2021. MMWR Morb Mortal Wkly Rep (2021) 70:1059–62. doi: 10.15585/mmwr.mm7031e2 PMC8367314 34351882

[157] Hacisuleyman E Hale C Saito Y Blachere NE Bergh M Conlon EG. Vaccine Breakthrough Infections With SARS-CoV-2 Variants. N Engl J Med (2021) 384:2212–8. doi: 10.1056/NEJMoa2105000 PMC8117968 33882219

[158] McCray Jr PB Pewe L Wohlford-Lenane C Hickey M Manzel L Shi L. Lethal Infection of K18-Hace2 Mice Infected With Severe Acute Respiratory Syndrome Coronavirus. J Virol (2007) 81:813–21. doi: 10.1128/JVI.02012-06 PMC1797474 17079315

[159] Monaco G Chen H Poidinger M Chen J de Magalhães JP Larbi A. flowAI: Automatic and Interactive Anomaly Discerning Tools for Flow Cytometry Data. Bioinformatics (2016) 32:2473–80. doi: 10.1093/bioinformatics/btw191 27153628

