# Supplementary material for: Time-Dependent Increase in Susceptibility and Severity of Secondary Bacterial Infections During SARS-CoV-2
Source: Front Immunol. 2022 May 12;13:894534. doi: 10.3389/fimmu.2022.894534 (PMC9134015; doi:10.3389/fimmu.2022.894534)
Supplement: Supplementary file 1 [file DataSheet_1.pdf]

## Supplemental Material

# Time-Dependent Increase in Susceptibility and Severity of Secondary Bacterial Infection during SARS-CoV-2 Infection

Amanda P. Smith[a], Evan P. Williams[b], Taylor R. Plunkett[b], Muneeswaran Selvaraj[c,†], Lindey C. Lane[e], Lillian Zalduondo[b], Yi Xue[b], Peter Vogel[f], Rudragouda Channappanavar[b,c,d,†], Colleen B. Jonsson[b,d,#], Amber M. Smith[a,b,d,#]

[a]Department of Pediatrics, University of Tennessee Health Science Center, Memphis, TN, USA
[b]Department of Microbiology, Immunology and Biochemistry, University of Tennessee Health Science Center, Memphis, TN, USA
[c]Department of Acute and Tertiary Care, University of Tennessee Health Science Center, Memphis, TN, USA
[d]Institute for the Study of Host-Pathogen Systems, University of Tennessee Health Science Center, Memphis, TN, USA
[e]College of Pharmacy, University of Tennessee Health Science Center, Memphis, TN, USA
[f]Animal Resources Center and Veterinary Pathology Core, St. Jude Children's Research Hospital, Memphis, TN, USA

[#]Address correspondence to amber.smith@uthsc.edu , cjonsson@uthsc.edu
[†]Current affiliation: Department of Veterinary Pathobiology, Oklahoma State University, Stillwater, OK, USA

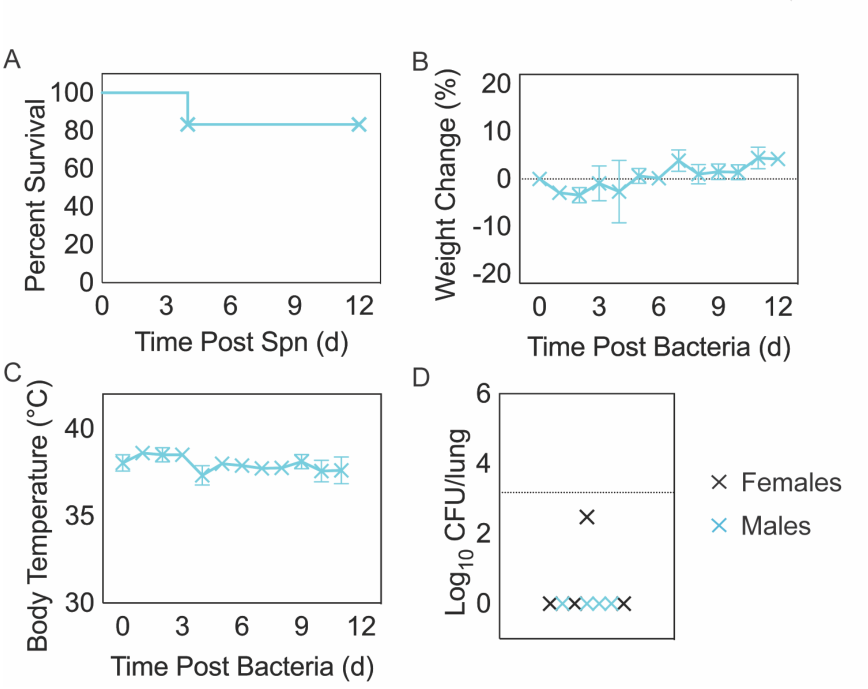


**Figure S1: *Streptococcus pneumoniae* infection in naïve K18-hACE2 mice.** Kaplan-Meier survival curve (A), percent weight loss (B), and temperature (C) of mice infected with $10^3$ CFU D39. Data are shown as the mean ± standard deviation (SD). Lung bacterial loads (CFU/lung) (D) in female (black) and male (cyan) mice infected with $10^3$ CFU D39 for 24 hours.

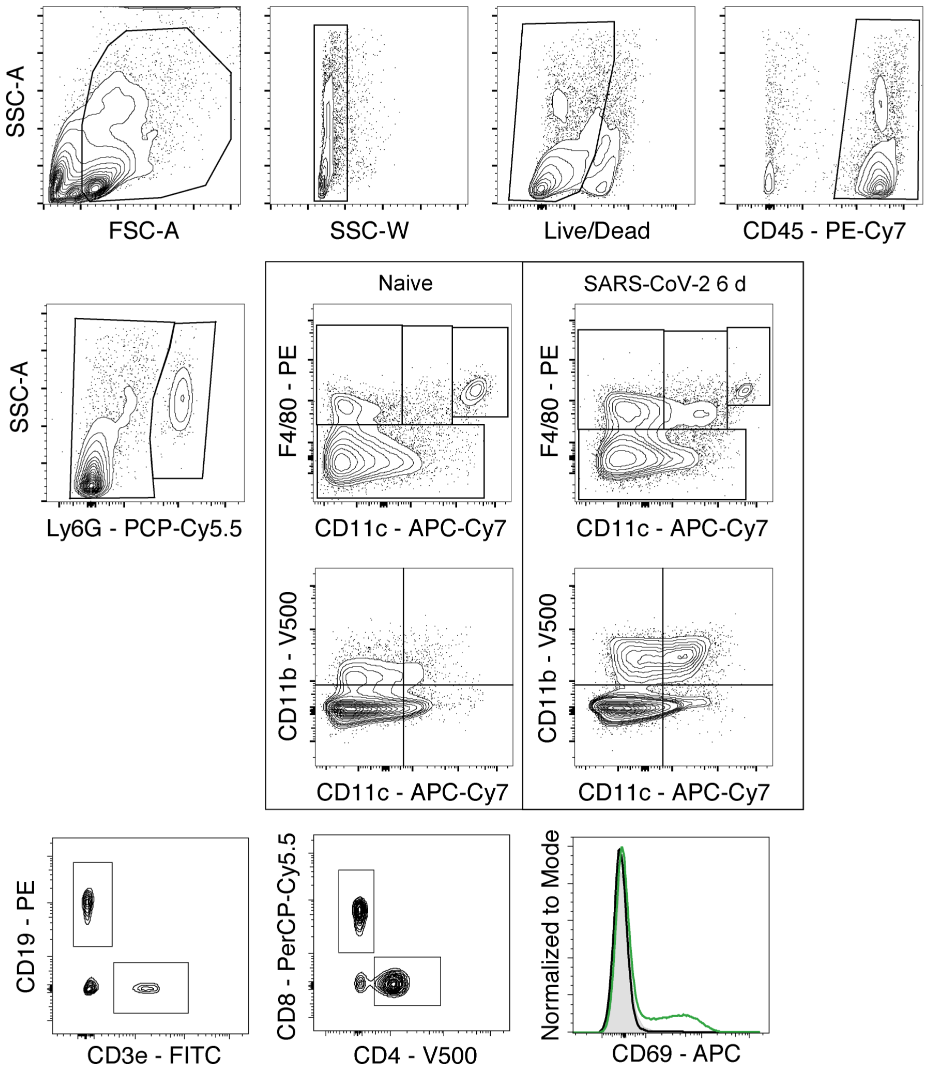


**Figure S2: Flow cytometry gating scheme for lung cell analysis.** Viable immune cells were first gated on forward scatter (FSC-A) and side scatter (SSC-A), as singlets, fixable viability dye negative, and $CD45^{+}$ (top row). Neutrophils ($Ly6G^{hi}$) were then gated and excluded from remaining parent populations. Monocyte and macrophage (MΦ) subsets were gated based on expression of CD11c and F4/80 with alveolar macrophages (AMΦ) sub-gated as $F4/80^{hi}CD11c^{hi}CD11b^{-}MHC\text{-}II^{low/-}$, inflammatory macrophages (iMΦ) as $F4/80^{hi}CD11c^{hi}CD11b^{+}MHC\text{-}II^{mid/hi}$, and additional subsets as $F4/80^{mid}CD11c^{mid}CD11b^{+}$ and $F4/80^{mid}CD11c^{-}CD11b^{+/-}$. Following MΦ exclusion, B cells were gated as $CD3e^{-}CD19^{+}$ and T cells were gated as $CD3e^{+}$ and subgated into $CD8^{+}$ T cells ($CD3e^{+}CD8^{+}CD4^{-}CD335^{-}$), $CD4^{+}$ T cells ($CD3e^{+}CD8^{-}CD4^{+}CD335^{-}$), and NK T cells ($CD3e^{+}CD335^{+}$). Natural killer (NK) cells were gated $CD3e^{-}CD19^{-}CD335^{+}$. Surface CD69 expression was used to quantify activation in all gated populations.

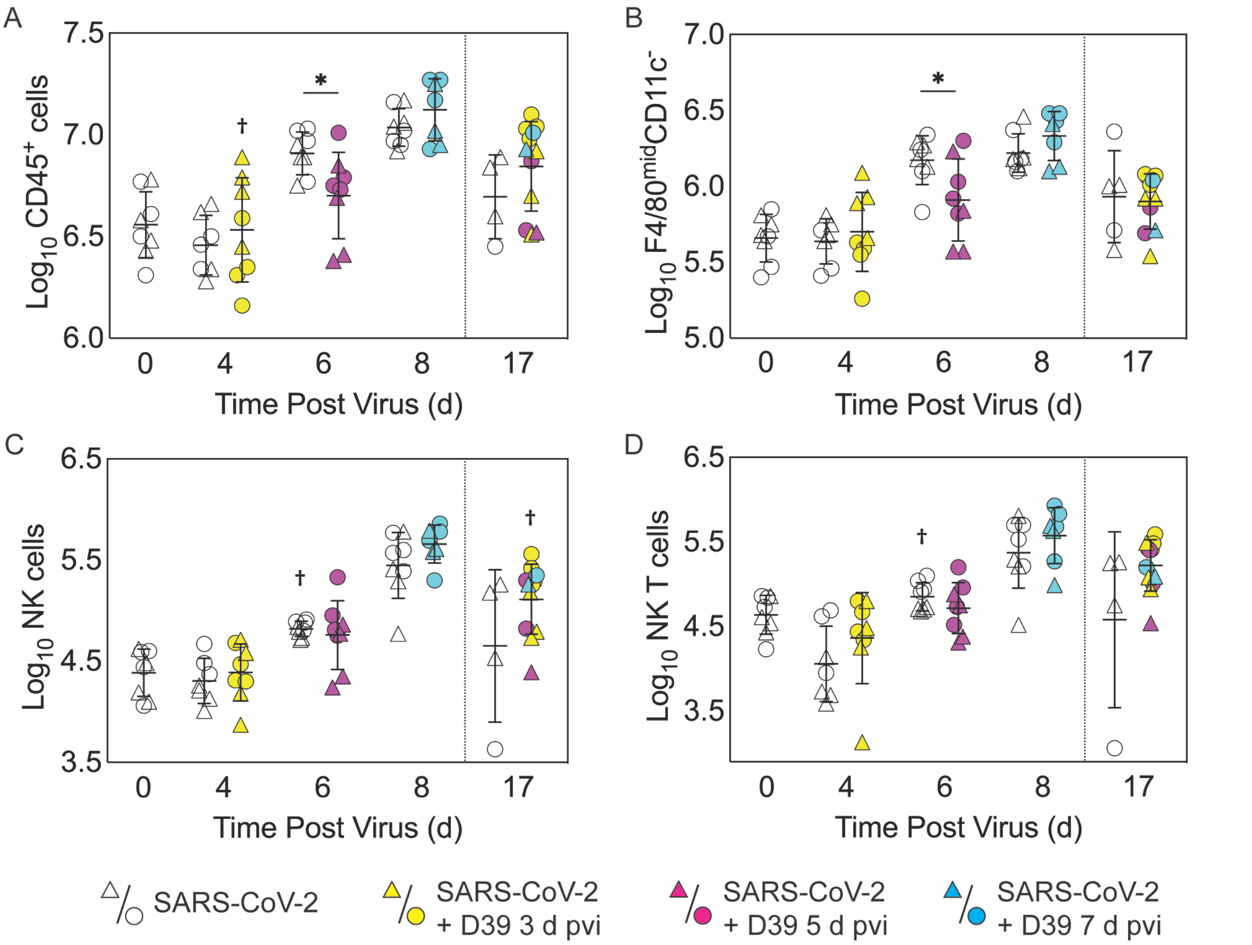


**Figure S3: Quantification of additional immune cell populations.** Total $CD45^+$ cells (A), $F4/80^{mid}CD11c^-CD11b^{+/-}$ cells (B), NK cells (C), and NK-T cells (D) in the lungs of female (circles) and male (triangle) mice infected with SARS-CoV-2 (250 PFU; open symbols) followed by $10^3$ CFU D39 at 3 d (yellow), 5 d (magenta), or 7 d (cyan) pvi. Each symbol represents a single mouse and the mean ± standard deviation (SD) are for combined male and female groups. Significant differences are indicated by *, $P < .05$ for comparisons between indicated groups and as †, $P < .05$ for differences between males and females within a group or between coinfection times within 17 d group. Plots depicting additional cells are shown in Figure 3 of the main text and the flow cytometry gating scheme is in Figure S2.

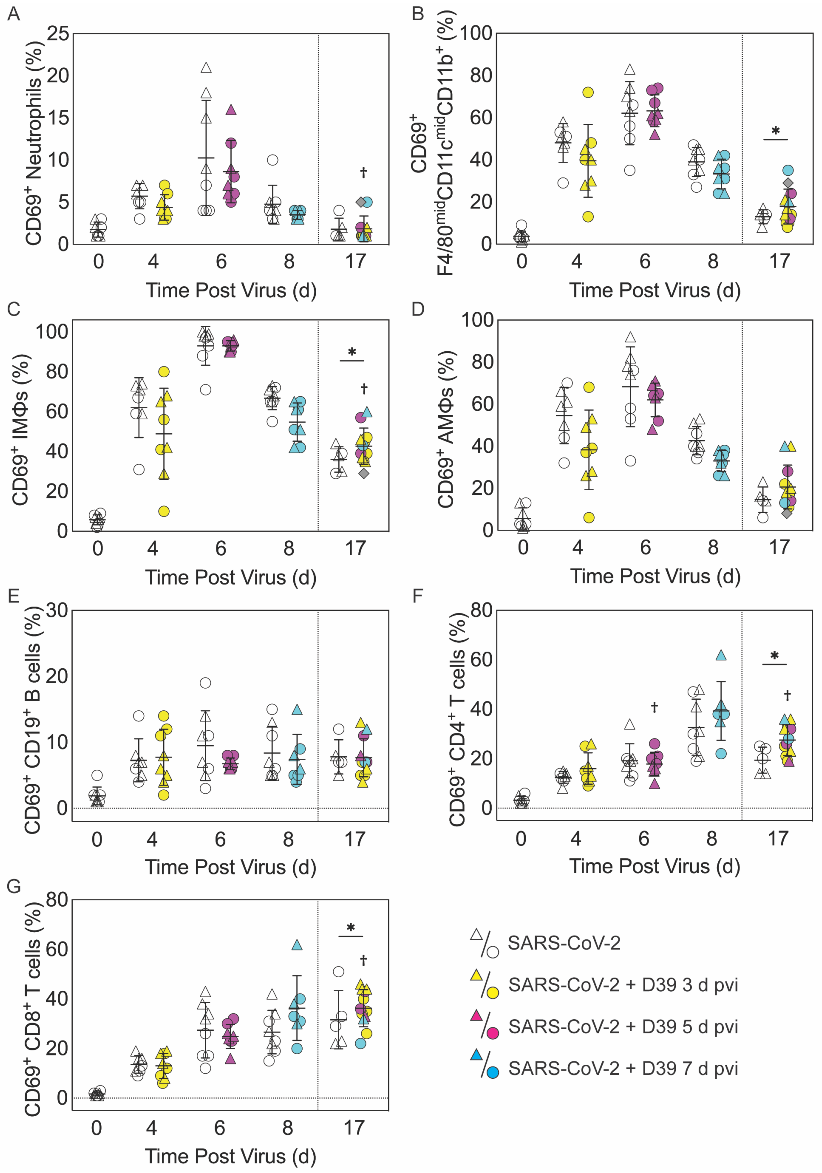


**Figure S4: Immune cell activation during SARS-CoV-2 infection and pneumococcal coinfection.** The percentage of CD69$^+$ neutrophils (A), F4/80$^{mid}$CD11c$^{mid}$CD11b$^+$ (B), iMΦ (F4/80$^{hi}$CD11c$^{hi}$CD11b$^+$MHC-II$^{mid/hi}$) (C) AMΦ (F4/80$^{hi}$CD11c$^{hi}$CD11b$^-$MHC-II$^{low/-}$) (D), CD19$^+$ B cells (E), CD4$^+$ T cells (F), and CD8$^+$ T cells (G) in the lungs of female (circles) and male (triangle) mice infected with SARS-CoV-2 (250 PFU; open symbols) followed by $10^3$ CFU D39 at 3 d (yellow), 5 d (magenta), or 7 d (cyan) pvi. Each symbol represents a single mouse and the mean ± standard deviation (SD) are for combined male and female groups. Significance comparisons were done using the absolute number of cells per lung and differences are indicated by *, *P* < .05 for comparisons between indicated groups and as †, *P* < .05 for differences between males and females within a group or between coinfection times within 17 d group.

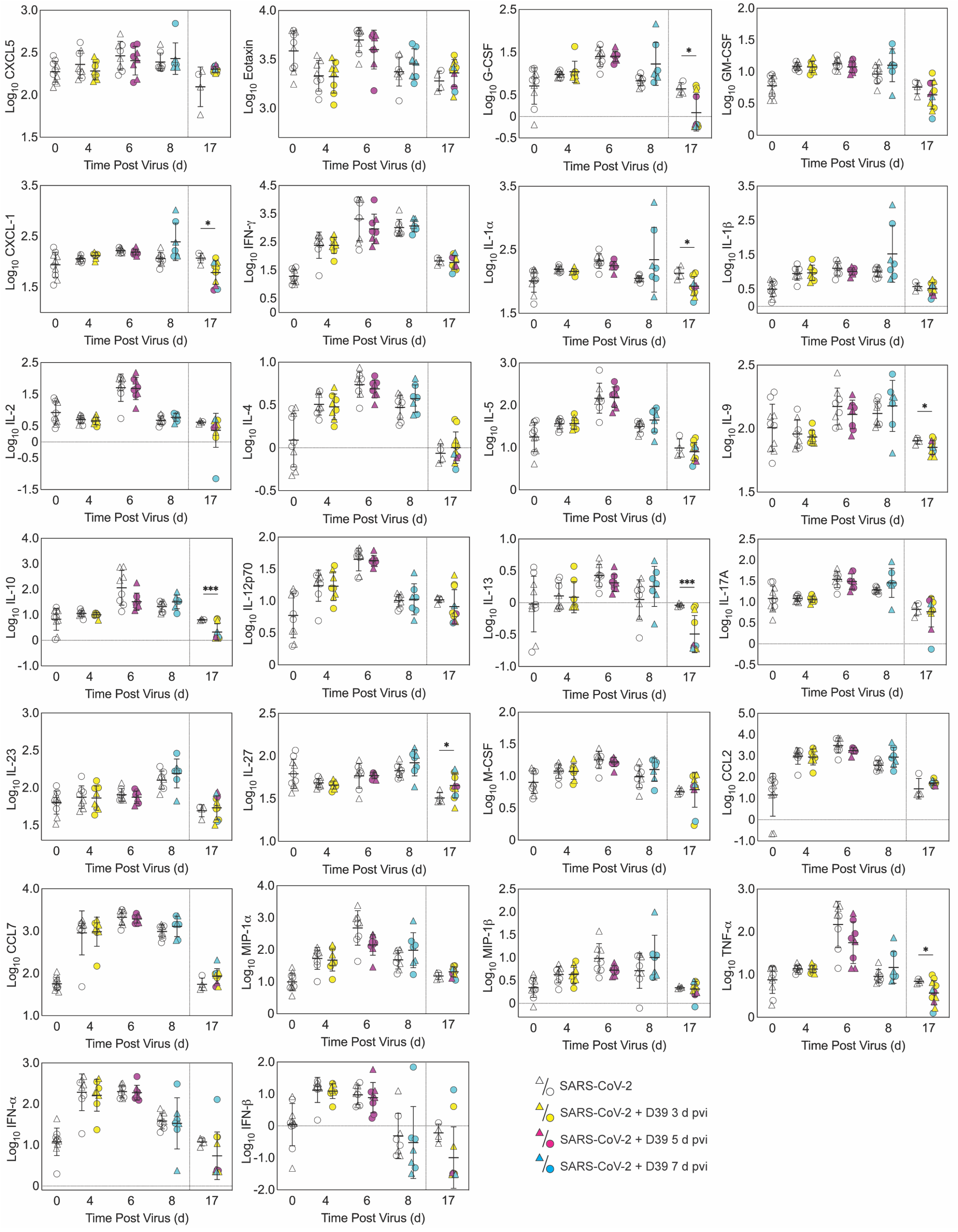


**Figure S5: Absolute quantities of pulmonary cytokines and chemokines during SARS-CoV-2 infection and pneumococcal coinfection.** The absolute picograms ($\log_{10}$) of cytokines and

chemokines in the lungs of female (circles) and male (triangle) mice infected with SARS-CoV-2 (250 PFU; open symbols) followed by $10^3$ CFU D39 at 3 d (yellow), 5 d (magenta), or 7 d (cyan) pvi. Each symbol represents a single mouse and the mean ± standard deviation (SD) are for combined male and female groups. Significant differences are indicated by *, $P < .05$; **, $P < .01$; and ***, $P < .001$ for comparisons between indicated groups. Plots depicting additional cytokine and chemokine quantities (absolute $\log_{10}$ picograms) are in Figure 4 of the main text and a heatmap representing the normalized quantity (average $\log_2$ change over naïve) is in Figure S6.

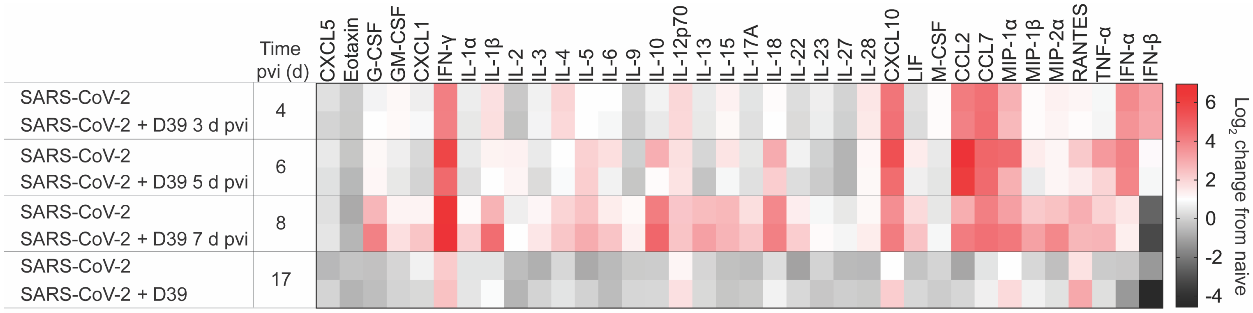


**Figure S6: Fold change over naïve of pulmonary cytokines and chemokines during SARS-CoV-2 infection and SARS-CoV-2-pneumococcal coinfection.** Heatmap representing the normalized quantity (average $\log_2$ change over naïve) of 36 cytokines and chemokines in the lungs of mice infected with SARS-CoV-2 (250 PFU) followed by $10^3$ CFU D39 at 3, 5, or 7 d pvi. Plots depicting absolute $\log_{10}$ picograms (pg) of measured cytokines and chemokines are in Figure 4 and Figure S5.
